# Identification of Selective ERRγ Inverse Agonists

**DOI:** 10.3390/molecules21010080

**Published:** 2016-01-12

**Authors:** Jina Kim, Chun Young Im, Eun Kyung Yoo, Min Jung Ma, Sang-Bum Kim, Eunmi Hong, Jungwook Chin, Hayoung Hwang, Sungwoo Lee, Nam Doo Kim, Jae-Han Jeon, In-Kyu Lee, Yong Hyun Jeon, Hueng-Sik Choi, Seong Heon Kim, Sung Jin Cho

**Affiliations:** 1 New Drug Development Center, Daegu-Gyeongbuk Medical Innovation Foundation, Daegu 41061, Korea; jina@dgmif.re.kr (J.K.); cyim@dgmif.re.kr (C.Y.I.); minjung@dgmif.re.kr (M.J.M.); ksb2014@dgmif.re.kr (S.-B.K.); turtulee@dgmif.re.kr (E.H.); jwchin@dgmif.re.kr (J.C.); hwanghy@dgmif.re.kr (H.H.); swlee@dgmif.re.kr (S.L.); namdoo@dgmif.re.kr (N.D.K.); 2 Leading-Edge Research Center for Drug Discovery and Development for Diabetes and Metabolic Disease, Kyungpook National University Hospital, Daegu 41404, Korea; tong-e@hanmail.net (E.K.Y.); ggoloo@hanmail.net (J.-H.J.); leei@knu.ac.kr (I.-K.L.); 3 Department of Internal Medicine, School of Medicine, Kyungpook National University, Daegu 41944, Korea; 4 Department of Nuclear Medicine, School of Medicine, Kyungpook National University, Daegu 41944, Korea; jeon9014@gmail.com; 5 National Creative Research Initiatives Center for Nuclear Receptor Signals and Hormone Research Center, School of Biological Sciences and Technology, Chonnam National University, Gwangju 61186, Korea; hsc@chonnam.ac.kr

**Keywords:** estrogen-related receptor gamma, inverse agonist, ADMET, GSK5182

## Abstract

GSK5182 (**4**) is currently one of the lead compounds for the development of estrogen-related receptor gamma (ERRγ) inverse agonists. Here, we report the design, synthesis, pharmacological and *in vitro* absorption, distribution, metabolism, excretion, toxicity (ADMET) properties of a series of compounds related to **4**. Starting from **4**, a series of analogs were structurally modified and their ERRγ inverse agonist activity was measured. A key pharmacophore feature of this novel class of ligands is the introduction of a heterocyclic group for A-ring substitution in the core scaffold. Among the tested compounds, several of them are potent ERRγ inverse agonists as determined by binding and functional assays. The most promising compound, **15g**, had excellent binding selectivity over related subtypes (IC_50_ = 0.44, >10, >10, and 10 μM at the ERRγ, ERRα, ERRβ, and ERα subtypes, respectively). Compound **15g** also resulted in 95% transcriptional repression at a concentration of 10 μM, while still maintaining an acceptable *in vitro* ADMET profile. This novel class of ERRγ inverse agonists shows promise in the development of drugs targeting ERRγ-related diseases.

## 1. Introduction

Estrogen-related receptors (ERRs), comprising ERRα, ERRβ, and ERRγ, are constitutively active nuclear receptors closely related to the estrogen receptors (ER), ERα and ERβ, with high levels of sequence identity [1]. They are primarily expressed in tissues such as the heart, brain, kidney, pancreas, placenta, and liver [2], and are known to play a pivotal role in the development of diseases such as cancer and various metabolic disorders [3]. Among the ERRs, ERRγ in particular has been shown to be involved in metabolic diseases such as type 2 diabetes mellitus, alcohol-induced oxidative stress, liver injury, and microbial infections caused by impaired hepatic gluconeogenesis [4,5], impaired hepatic insulin signaling [6], and impaired iron metabolism [7].

**Figure 1:**
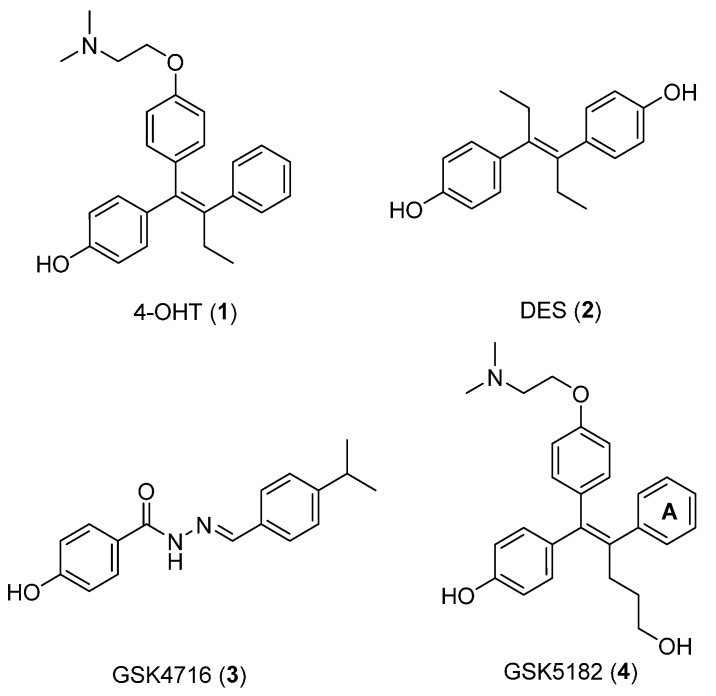
The ERRγ ligands. 4-OHT (**1**): ERα antagonist/ERRγ inverse agonist; DES (**2**): ERα agonist/ERRγ inverse agonist; GSK4716 (**3**): selective ERRγ agonist; GSK5182 (**4**): selective ERRγ inverse agonist.

Based on these findings, ERRγ has emerged as a promising target in the treatment of certain metabolic disorders and cancers. However, despite its biological versatility, only a handful of studies examining ERRγ ligands that modulate downstream signals have been performed in the past two decades. Consequently, to date, there are few synthetic compounds targeting ERRγ with interesting pharmacological activity (Figure 1). The first synthetic compounds, 4-hydroxytamoxifen (**1**, 4-OHT) [8] and diethylstilbestrol (**2**, DES) [9], were identified as an ERα antagonist/ERRγ inverse agonist and an ERα agonist/ERRγ inverse agonist, respectively. In addition, the acyl hydrazine, GSK4716 (**3**), was reported as an ERRγ agonist with improved binding selectivity over the classical ER, yet expressed mixed ERRγ/ERRβ functional activities in cell-based assay systems [10]. This mandated the development of a more selective inverse agonist for ERRγ; therefore, GSK5182 (**4**) was developed as a 4-OHT (**1**) analog that showd better selectivity *vs.* the structurally related nuclear receptor ERα [11].

Several lines of evidence have revealed that the ERRγ inverse agonist **4** alleviates diabetes through the inhibition of hepatic gluconeogenesis in a PGC-1α-dependent manner [5]. Additionally, **4** has antimicrobial effects by reducing ERRγ-mediated hepcidin mRNA expression [7]. More recently, it was reported that **4** enhances the responsiveness of radioiodine therapy by modulating sodium iodide symporter (NIS) function in ATC cells via the regulation of ERRγ and the MAP kinase signaling pathway [12].

In spite of these findings, the discovery of newly synthesized ERRγ ligands displaying better selectivity and potency is required not only to better understand the essential biological roles of ERRγ but also to eventually develop novel therapeutic agents. Therefore, the purpose of this study was to identify new ERRγ inverse agonists with increased potency and selectivity than those currently available.

In this article, we describe the discovery and *in vitro* binding and functional characterization of novel ERRγ selective inverse agonists bearing comparable drug-like properties to those of **4** on the basis of *in vitro* ADMET considerations. Inspired by the early docking studies suggesting that the A-ring of **4** had structural binding flexibility in the ERRγ binding pocket, our initial strategy was focused on synthesizing A-ring analogs of **4**. Thus, the lead optimization efforts were focused on modification of the A-ring phenyl group with various hydroxyaryl and heterocyclic substituents, in order to improve the *in vitro* ADMET profile good enough for subsequent *in vivo* assessment.

## 2. Results and Discussion

### 2.1. Candidate Synthesis

**Scheme 1:**
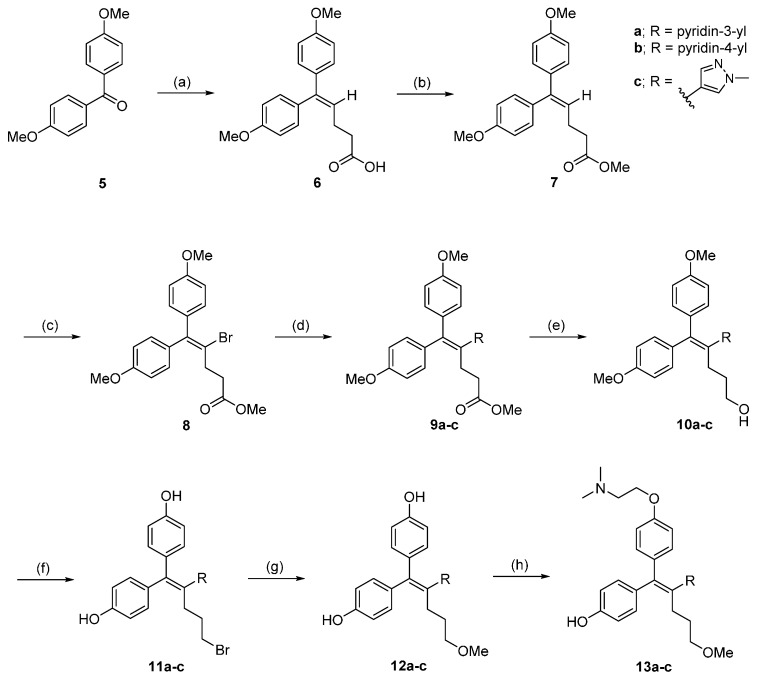
Compounds **13a**–**c** were synthesized according to the well-established methodology. *Reagents and conditions*: (**a**) (3-Carboxypropyl)triphenylphosphonium bromide, NaH, DMSO/THF, 0 °C to rt; (**b**) SOCl_2_, MeOH, 70 °C; (**c**) CuBr_2_, CCl_4_, 80 °C; (**d**) R-B(OH)_2_ or R-Bpin, Pd(dppf)Cl_2_·CH_2_Cl_2_, K_3_PO_4_, DMF/H_2_O, 82 °C; (**e**) LiAlH_4_, THF, 0 °C; (**f**) BBr_3_, CH_2_Cl_2_, 80 °C; (**g**) NaOCH_3_, MeOH, 0 °C; (**h**) 2-Chloro-*N*,*N*-dimethylethylamine hydrochloride, K_2_CO_3_, acetone/H_2_O, 63 °C.

Compounds **13a**–**c** were synthesized according to the well-established methodology detailed in Scheme 1. Starting from the commercially available compound, 4,4′-dimethoxybenzophenone, the key intermediate **8** was prepared in three steps by employing Wittig chemistry [13] with (3-carboxypropyl)triphenylphosphonium bromide followed by methyl esterification and bromo substitution to ethylene hydrogen. Suzuki cross-coupling between **8** and the corresponding boronic acids/esters was then performed to attach various aromatic groups to the A-ring (**9a**–**c**). Compounds **11a**–**c** were derived by reduction of the methyl ester group with LiAlH_4_, which were subsequently treated with BBr_3_ to give compounds **12a**–**c**. A Williamson ether synthesis was carried out with 2-chloro-*N*,*N*-dimethylethylamine to provide compounds **13a**–**c** after further purification using preparative HPLC from a mixture of E/Z isomers and undesired di-alkylated product (Scheme 1). The bromo-trisubstituted ethylene derivative, **8**, was also a very useful intermediate in the preparation of other analogs. Removal of **8**’s methyl group with BBr_3_ and a Mitsunobu reaction with corresponding alcohols gave the *E*/*Z* mixture of compound **14** with typical *E*:*Z* ratios of 1:1. Their *E*/*Z* characterization was determined by HSQC, HMBC and 2D NOESY measurements after separation on column chromatography (Supplementary Materials; Figures S1–S8) and only the *Z* form (**14**) among those was applied for the next reaction.

**Scheme 2:**
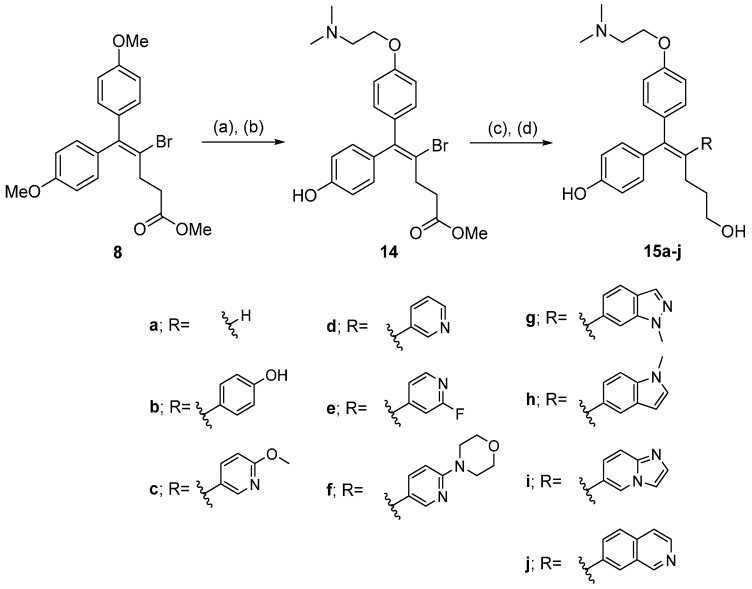
Compounds **15a**–**j** were prepared using a cross-coupling reaction between **14** and the corresponding boronic acids/esters, along with reduction of the methyl ester group with LiAlH_4_ or DIBAL-H. *Reagents and conditions*: (**a**) BBr_3_, CH_2_Cl_2_, 80 °C; (**b**) 2-(Dimethylamino)ethanol, PPh_3_, DIAD, CH_2_Cl_2_; (**c**) R-B(OH)_2_ or R-Bpin, Pd(dppf)Cl_2_·CH_2_Cl_2_, 2M Na_2_CO_3_, DMF, 85 °C; (**d**) LiAlH_4_, THF, 0 °C.

**Scheme 3:**
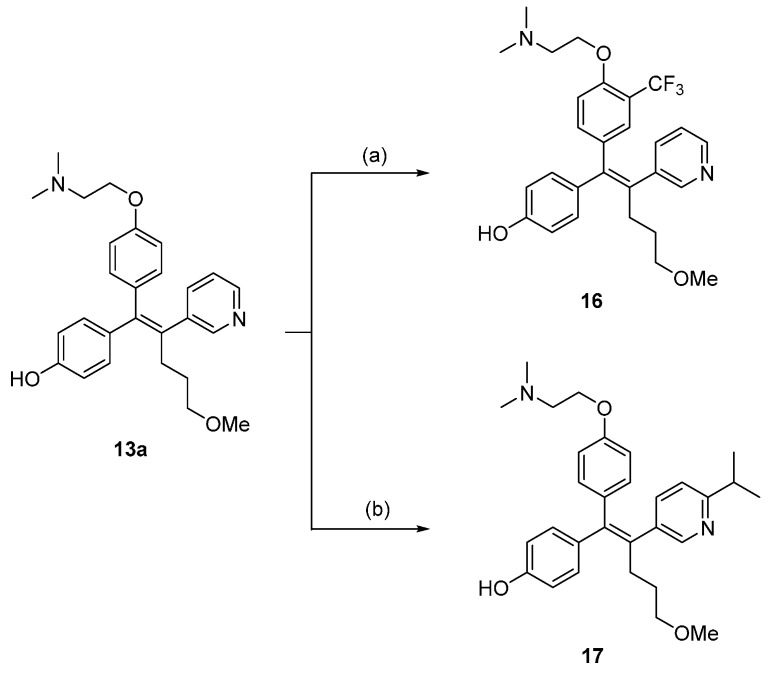
Compounds **16** and **17** were synthesized by direct trifluoromethylation and isopropylation of **13a**. *Reagents and conditions*: (**a**) Zinc trifluoromethanesulfinate, TFA, *tert*-Butyl hydroperoxide, CH_2_Cl_2_/H_2_O, 0 °C to rt; (**b**) Zinc isopropylsulfinate, TFA, *tert*-Butyl hydroperoxide, DMSO, 0 °C to rt.

Compounds **15a**–**j** were prepared using a cross-coupling reaction between **14** and the corresponding boronic acids/esters, along with reduction of the methyl ester group with LiAlH_4_ or DIBAL-H (Scheme 2). We also synthesized **16** and **17** by direct trifluoromethylation and isopropylation of **13a** (Scheme 3) [14].

### 2.2. Binding Assays

Initials creening was conducted using a well established TR-FRET based ERRγ binding assay as described in the Experimental section [15,16,17] at a concentration of 10 μM. Serial dilutions of the synthesized compounds were used to calculate IC_50_ values for the binding affinity with ERRγ. The results of these experiments are summarized in Table 1. Compound **4** was used as a positive control for comparison purposes.

**Table 1:** ERRγ binding affinity screening of 16 analogs. 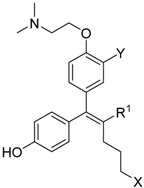

Compounds	R^1^	X	Y	ERRγ, IC_50_ (μM) ^a^
**4 (GSK5182)**	Ph	–OH	–H	0.11
**13a**	pyridin-3-yl	–OMe	–H	0.99
**13b**	pyridin-4-yl	–OMe	–H	>10
**13c**	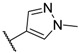	–OMe	–H	>10
**15a**	H	–OH	–H	>10
**15b**	4-OH-Ph	–OH	–H	2.32
**15c**	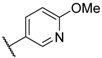	–OH	–H	>10
**15d**	pyridin-3-yl	–OH	–H	3.10
**15e**	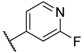	–OH	–H	5.35
**15f**	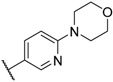	–OH	–H	>10
**15g**	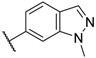	–OH	–H	0.44
**15h**	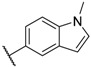	–OH	–H	>10
**15i**	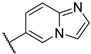	–OH	–H	>10
**15j**	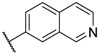	–OH	–H	4.71
**16**	pyridin-3-yl	–OMe	–CF_3_	>10
**17**	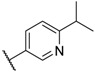	–OMe	–H	>10

^a^ IC_50_ value of binding inhibition of ERRγ binding activity; Abbreviations: Ph, phenyl; –OMe, methoxy.

While exploring the effects of R1 substitutions on binding affinity in the screening campaign of compounds **13a**–**17** in Table 1, we noticed that the A-ring is essential because the substitution of hydrogen as seen in compound **15a**, dramatically reduces ERRγ binding affinity. The presence of an alcohol para to the phenyl ring also poses a negative effect on binding affinity as seen in compound **15b**. Although introduction of a substituted heterocyclic system (compounds **15c**–**f**) led to a significant loss of affinity, while among the bicyclic system-adopted compounds **15g**–**j,** an analog with the Me-indazole group (compound **15g**) displayed decent binding affinity.

Incorporation of a methoxy group as a terminal –OH substitution resulted in significant loss of affinity in several heteroaryl-bearing compounds (**13b**,**c**, and **17**). The –OMe series showed no affinity at the maximum concentration of the assay (10 μM); the only notable difference occurred with compound **13a**,which had a slightly improved affinity (IC_50_ = 0.99 μM at the ERRγ). Furthermore, by comparing compound **13a** with **16**, we determined that –CF_3_ substitution to the *ortho* position of the *N*,*N*-dimethylaminoethoxy group resulted in no affinity at the screening concentration.

### 2.3. Functional Activity Assays

**Figure 2:**
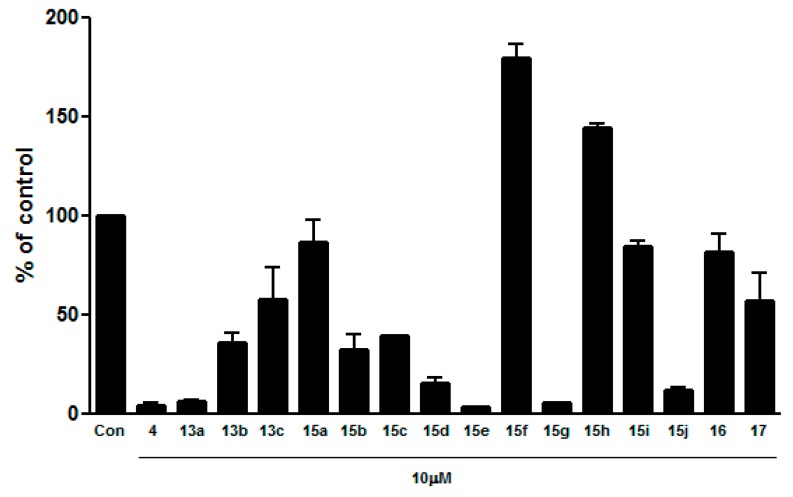
Cell based reporter-gene assay screening of 16 analogs ERRγ binding activity in cell was measured by luciferase activity using co-transfection system with GAL4-ERRγ and pFR-luciferase reporter gene.

Along with the binding assay campaign, functional activity was determined in order to validate the utility of the binding affinity in a cellular context as described in the Experimental [5]. The results of these experiments are summarized in Figure 2. Compounds that displayed poor activity in the binding assay (IC_50_ > 10 μM) were found to be moderate (**13b**,**c**, **15c**, **17**) or to inactivate (**15a**,**i**, **16**); transcriptional repression of **13b**,**c**, **15c**, **17** at the screening concentration of 10 μM is around 50% and that of **15a**,**i**, **16** is around 0%. However, compounds **15f**,**h** deviated from the trend established by other compounds with poor activity in the binding assay, showing partial agonistic activity in the cellular system. Nonetheless, compounds **13a**, **15e**,**g** displayed comparable regression of the ERRγ transcriptional activity compared with the control ligand, **4**.

### 2.4. Molecular Modeling Study

**Figure 3:**
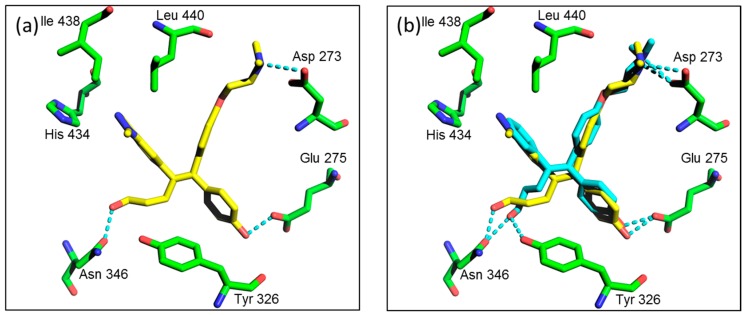
Proposed binding model of compound **15g**. (**a**) The protein structure originated from the X-ray structure of its complex with **4** (pdb code: 2ewp). ERRγ is shown in a green color and the hydrogen bond interactions with compound **15g** is shown as dotted lines; (**b**) Comparison of the proposed binding mode of compound **15g** and **4** with ERRγ.

Next, we performed a docking simulation analysis in order to suggest a molecular model describing the binding of compound **15g** to ERRγ. The docking study revealed that compound **15g** may bind to the active site via hydrogen bonding with Asp 273, Glu 275, and Asn 346 of ERRγ, respectively. The docking study results suggest that compound **15g** overlaps the active site of ERRγ in a similar manner as **4** (Figure 3).

### 2.5. Selectivity and ADMET Consideration

Based on results from the *in vitro* screening tests, we chose compound **15g** for additional studies, including a binding selectivity test, cytochrome P450 screening, determination of metabolic stability in liver microsomes, and hERG inhibition. Compound **4**, with high binding affinity for ERRγ and a good selectivity profile over the closely related receptors ERRα, ERRβ and ERα, served as the positive control.

The binding selectivity profile of **15g** was comparable to **4**, with no observable affinity over ERRα, ERRβ and ERα at the maximum concentration tested (10 μM). CYP inhibition was determined against five major subtypes, including 1A2, 2C9, 2C19, 2D6, and 3A4, which are primarily involved in drug metabolism. Compound **15g** maximally inhibited the CYP 2C9 subtype by 17% at a concentration of 10 μM and showed nearly no inhibition against other subtypes. In comparison, compound **4** generally inhibits the CYP enzymes by 15%–27% (Table 2).

**Table 2:** *In vitro* binding and functional characterization and in vitro ADMET profile of **15g**.

Componds	Binding Assay, IC_50_ (μM)				ERRγ Functional Assay at 10 μM (% of Control)	CYP Inhibition (% of Control)					MS (%)				hERG IC_50_ (μM)
	ERRγ	ERRα	ERRβ	ERα		1A2	2C9	2C19	2D6	3A4	Human	Dog	Rat	Mouse	
**4 (GSK5182)**	0.110	>10	>10	2	6.6	84	73	78	82	83	43	9.6	26	6.8	>30
**15g**	0.440	>10	>10	>10	5.4	>100	83	95	99	88	32	46	34	42	>30

Compound **15g** had improved metabolic stability overall with greater than 30% remaining in four types of microsomes, compared with less than 10% of compound **4** remaining in a both dog and mouse microsome. No hERG inhibition was detected for compound **15g** or compound **4** (IC_50_ values >30 μM) (Table 2).

## 3. Experimental Section

### 3.1. General Information

All NMR experiments were carried out using an Avance III 400 MHz NMR spectrometer equipped with a 5-mm broadband-observed probe head (Bruker, Billerica, MA, USA). The NMR spectrum optimization was conducted using the Bruker Topspin 3.1 software, and all parameters were set in it. The compounds were dissolved in CDCl_3_ or *d*-MeOH and the spectra were acquired at 25 °C. In the 2D experiments, the ^1^H chemical shifts (δ) were referenced to the residual solvent peak: *d*-MeOH, δ = 3.31 (^1^H), δ = 49.2 (^13^C). ^13^C-heteronuclear single-quantum-correlated (HSQC) and ^13^C-heteronuclear multiple-bond-correlated (HMBC) spectra were recorded for the assignment of carbon and proton resonances. The ^1^H-^1^H 2D NOESY with mixing times of 300 ms, 600 ms were performed to obtain information on proton distance. Mass spectra were measured in positive electrospray ionization (ESI) mode on LCMS-2020 system (Shimadzu, Tokyo, Japan). Column chromatography was performed using a CombiFlash^®^ Rf system with RediSep^®^ Rf (Teledyne Isco, Lincoln, NE, USA). For the final compounds, further purification was performed by preparative HPLC on Kinetex^®^ 5 μm Biphenyl 100 Å (GX-281 HPLC system, Gilson, Middleton, WI, USA; column tube: 250 mm × 21.2 mm ID) with ACN/H_2_O as eluent. The purity of the target compounds was determined to be >95% by analytical HPLC using dual different wavelength UV detector. Starting materials were obtained from Aldrich (St. Louis, MO, USA), or Alfa Aesa (Ward Hill, MA, USA). Solvents were obtained from Fisher Scientific (Hampton, NH, USA) or Aldrich and were used without further purification unless noted otherwise.

### 3.2. Chemistry

#### 3.2.1. Synthesis of 5,5-bis(4-Methoxyphenyl)pent-4-enoic Acid (**6**)

In an inert atmosphere, NaH (2.0 g, 49.5 mmol) was added in dry DMSO (50 mL). The mixture was stirred at 70 °C for 1 h. The mixture was cooled to 20 °C and 4-(bromotriphenylphosphoranyl)-butanoic acid (8.5 g, 19.8 mmol) was added in several portions over 5 min. The red solution was stirred at 20 °C for 15 min, then a solution of bis(4-methoxyphenyl)methanone (6.0 g, 24.8 mmol) in dry THF (33 mL) was added at such a rate. The mixture was stirred at rt for 19 h, then it was diluted with ice water and extracted with CH_2_Cl_2_. The organic extracts were discarded and the aqueous layer was acidified with 12 N HCl and extracted with CH_2_Cl_2_. The organic layer was washed with brine (2 times) and dried over MgSO_4_. The crude compound was purified by column chromatography to give the desired product (3.1 g, 50% yield).

#### 3.2.2. Synthesis of Methyl 5,5-bis(4-Methoxyphenyl)pent-4-enoate (**7**)

To a solution of 5,5-bis(4-methoxyphenyl)pent-4-enoic acid (3.1 g, 9.92 mmol) in MeOH (60 mL) stirred at 0 °C, SOCl_2_ (0.8 mL, 10.7 mmol) was added dropwise. The reaction mixture was heated to reflux for 2 h. The reaction mixture was removed and added water. The mixture was extracted with EtOAc and washed with aq. K_2_CO_3_, brine and dried over MgSO_4_. The solvent was removed under reduced pressure which was used for the next step without further purification (2.9 g, 91% yield).

#### 3.2.3. Methyl 4-bromo-5,5-bis(4-Methoxyphenyl)pent-4-enoate (**8**)

To a solution methyl 5,5-bis(4-methoxyphenyl)pent-4-enoate (4.0 g, 12.4 mmol) in CCl_4_ (45 mL) was added copper(II) bromide (6.91 g, 30.9 mmol) at rt. The reaction mixture was heated to reflux at 83 °C for 12 h, cooled to room temperature and filtered off through a Celite pad. The solvent was removed and reaction mixture was dissolved in EtOAc. The mixture was washed with water, brine and dried over MgSO_4_. The crude compound was purified by column chromatography to give the brown oil product (4.5 g, 89% yield).

#### 3.2.4. General Synthetic Procedure for **9a**–**c**

To a solution **8** (1 eq) in DMF/H_2_O (50:1) was added corresponding boronic esters or boronic acid (1.2 eq), PdCl_2_(dppf)·CH_2_Cl_2_ (0.1 eq) and K_3_PO_4_ (3 eq) at rt. The reaction mixture was heated to 82 °C for 12 h. The reaction mixture was cooled to rt and was quenched by adding water. The mixture was extracted with EtOAc and washed with brine and dried over MgSO_4_, filtered, and concentrated. The residue was purified by column chromatography to give the desired product.

*Methyl 5,5-bis(4-methoxyphenyl)-4-(pyridin-3-yl)pent-4-enoate* (**9a**). (2 g, 72% yield). ^1^H-NMR (400 MHz, CDCl_3_) δ 8.34 (m, 2H), 7.45 (dt, *J* = 6.3, 1.4 Hz, 1H), 7.12 (m, 3H), 6.89 (d, *J* = 6.9 Hz, 2H), 6.76 (d, *J* = 7.0 Hz, 2H), 6.56 (d, *J* = 7.0 Hz, 2H), 3.83 (s, 3H), 3.68 (s, 3H), 3.56 (s, 3H), 2.83 (m, 2H), 2.32 (m, 2H).

*Methyl 5,5-bis(4-methoxyphenyl)-4-(pyridin-4-yl)pent-4-enoate* (**9b**). (0.8 g, 87% yield). ^1^H-NMR (400 MHz, CDCl_3_) δ 8.40 (d, *J* = 6.0 Hz, 2H), 7.12 (d, *J* = 8.8 Hz, 2H), 7.03 (d, *J* = 6.0 Hz, 2H), 6.89 (d, *J* = 8.4 Hz, 2H), 6.76 (d, *J* = 8.8 Hz, 2H), 6.58 (d, *J* = 8.8 Hz, 2H), 3.83 (s, 3H), 3.70 (s, 3H), 3.57 (s, 3H), 2.82 (m, 2H), 2.30 (m, 2H).

*Methyl 5,5-bis(4-methoxyphenyl)-4-(1-methyl-1H-pyrazol-4-yl)pent-4-enoate* (**9c**). (0.7 g, 72% yield). ^1^H-NMR (400 MHz, CDCl_3_) δ 7.07 (d, *J* = 7.0 Hz, 2H), 7.03 (s, 1H), 6.98 (d, *J* = 7.0 Hz, 2H), 6.87 (s, 1H), 6.84 (d, *J* = 7.0 Hz, 2H), 6.73 (d, *J* = 7.0 Hz, 2H), 3.80 (s, 3H), 3.76 (s, 3H), 3.75 (s, 3H), 3.61 (s, 3H), 2.71 (m, 2H), 2.46 (m, 2H).

#### 3.2.5. General Synthetic Procedure for **10a**–**c**

To a solution of 1 M LiAlH_4_ solution in THF (1.5 eq) stirred at 0 °C, **9a**–**c** (1 eq) in THF was added dropwise. The solution was stirred at 0 °C for 1 h and was quenched by adding H_2_O/5 N NaOH/H_2_O (1:1:3). The reaction mixture was filtered off through celite pad, washed with EtOAc and concentrated in vacuo. The crude compound was purified by column chromatography to give the desired product.

*5,5-bis(4-Methoxyphenyl)-4-(pyridin-3-yl)pent-4-en-1-ol* (**10a**). (0.6 g, 85% yield). ^1^H-NMR (400 MHz, CDCl_3_) δ 8.35 (d, *J* = 1.1 Hz, 1H), 8.33 (d, *J* = 3.8 Hz, 1H), 7.43 (dt, *J* = 6.3, 1.4 Hz, 1H), 7.16 (d, *J* = 7.0 Hz, 2H), 7.10 (dd, *J* = 6.2, 3.9 Hz, 1H), 6.89 (d, *J* = 7.0 Hz, 2H), 6.77 (d, *J* = 7.1 Hz, 2H), 6.56 (d, *J* = 7.0 Hz, 2H), 3.83 (s, 3H), 3.68 (s, 3H), 3.54 (t, *J* = 5.2 Hz, 2H), 2.56 (m, 2H), 1.60 (m, 2H).

*5,5-bis(4-Methoxyphenyl)-4-(pyridin-4-yl)pent-4-en-1-ol* (**10b**). (0.6 g, 74% yield). ^1^H-NMR (400 MHz, CDCl_3_) δ 8.37 (d, *J* = 6.0 Hz, 2H), 7.14 (d, *J* = 8.8 Hz, 1H), 7.03 (d, *J* = 6.0 Hz, 2H), 6.89 (d, *J* = 8.8 Hz, 2H), 6.78 (d, *J* = 8.4 Hz, 2H), 6.58 (d, *J* = 8.8 Hz, 2H), 3.83 (s, 3H), 3.70 (s, 3H), 3.55 (t, *J* = 6.8 Hz, 2H), 2.55 (m, 2H), 1.59 (m, 2H).

*5,5-bis(4-Methoxyphenyl)-4-(1-methyl-1H-pyrazol-4-yl)pent-4-en-1-ol* (**10c**). (0.9 g, 73% yield). ^1^H-NMR (400 MHz, CDCl_3_) δ 7.11 (d, *J* = 6.9 Hz, 2H), 7.07 (s, 1H), 6.99 (d, *J* = 7.0 Hz, 2H), 6.85 (d, *J* = 7.0 Hz, 2H), 6.83 (s, 1H), 6.74 (d, *J* = 7.0 Hz, 2H), 3.80 (s, 3H), 3.76 (s, 3H), 3.74 (s, 3H), 3.58 (m, 2H), 2.46 (m, 2H), 1.75 (m, 2H).

#### 3.2.6. General Synthetic Procedure for **11a**–**c**

To a solution of **10a**–**c** (1 eq) in CH_2_Cl_2_ stirred at 0 °C, 1 M BBr_3_ solution in CH_2_Cl_2_ (10 eq) was added dropwise. The solution was refluxed at 80 °C for 12 h and was quenched by adding H_2_O at 0 °C. The reaction mixture was diluted with EtOAc and washed with H_2_O and brine. The organic layer was dried MgSO_4_ and concentrated *in vacuo*. The residue was purified by column chromatography to give the desired product.

*4,4′-(5-Bromo-2-(pyridin-3-yl)pent-1-ene-1,1-diyl)diphenol* (**11a**). (0.7 g, 86% yield). ^1^H-NMR (400 MHz, DMSO-*d*_6_) δ 9.53 (s, OH), 9.35 (s, OH), 8.59 (d, *J* = 4.3 Hz, 1H), 8.51 (s, 1H), 8.24 (d, *J* = 6.5 Hz, 1H), 7.81 (t, *J* = 6.2 Hz, 1H), 7.02 (d, *J* = 6.8 Hz, 2H), 6.77 (d, *J* = 6.8 Hz, 2H), 6.66 (d, *J* = 5.2 Hz, 2H), 6.49 (d, *J* = 6.9 Hz, 2H), 3.44 (t, *J* = 5.2 Hz, 2H), 2.64 (m, 2H), 1.82 (m, 2H).

*4,4′-(5-Bromo-2-(pyridin-4-yl)pent-1-ene-1,1-diyl)diphenol* (**11b**). (0.7 g, 99% yield). ^1^H-NMR (400 MHz, DMSO-*d*_6_) δ 9.59 (s, OH), 9.47 (s, OH), 8.61 (d, *J* = 4.6 Hz, 2H), 7.60 (d, *J* = 3.5 Hz, 2H), 7.02 (d, *J* = 7.7 Hz, 2H), 6.78 (d, *J* = 6.8 Hz, 2H), 6.66 (d, *J* = 6.9 Hz, 2H), 6.50 (d, *J* = 6.8 Hz, 2H), 3.43 (t, *J* = 5.2 Hz, 2H), 2.66 (m, 2H), 1.79 (m, 2H).

*4,4′-(5-Bromo-2-(1-methyl-1H-pyrazol-4-yl)pent-1-ene-1,1-diyl)diphenol* (**11c**). (0.2 g, 39% yield). ^1^H-NMR (400 MHz, DMSO-*d*_6_) δ 7.27 (s, 1H), 6.92 (d, *J* = 6.7 Hz, 2H), 6.81 (d, *J* = 6.7 Hz, 2H), 6.71 (s, 1H), 6.69 (d, *J* = 7.0 Hz, 2H), 6.62 (d, *J* = 6.7 Hz, 2H), 3.68 (s, 3H), 3.44 (t, *J* = 5.3 Hz, 2H), 2.41 (m, 2H), 1.91 (m, 2H).

#### 3.2.7. General Synthetic Procedure for **12a**–**c**

To a solution of **11a**–**c** (1 eq) in MeOH stirred at 0 °C, 5 M NaOCH_3_ solution in MeOH (10 eq) was added dropwise. The solution was stirred at rt for 12 h and quenched by adding 6 N HCl at 0 °C. The reaction mixture was diluted with MeOH and filtered. The residue was purified by column chromatography to give the desired product.

*4,4′-(5-Methoxy-2-(pyridin-3-yl)pent-1-ene-1,1-diyl)diphenol* (**12a**). (0.4 g, 90% yield). ^1^H-NMR (400 MHz, DMSO-*d*_6_) δ 9.53 (s, OH), 9.43 (s, OH), 8.60 (d, *J* = 5.6 Hz, 1H), 8.51 (s, 1H), 8.23 (d, *J* = 8.4 Hz, 1H), 7.81 (dd, *J* = 8.0, 5.6 Hz, 1H), 7.02 (d, *J* = 8.4 Hz, 2H), 6.78 (d, *J* = 8.8 Hz, 2H), 6.65 (d, *J* = 8.4 Hz, 2H), 6.49 (d, *J* = 11.4 Hz, 2H), 3.21 (t, *J* = 6.0 Hz, 2H), 3.17 (s, 3H), 2.55 (m, 2H), 1.50 (m, 2H).

*4,4′-(5-Methoxy-2-(pyridin-4-yl)pent-1-ene-1,1-diyl)diphenol* (**12b**). (0.4 g, 82% yield). ^1^H-NMR (400 MHz, DMSO-*d*_6_) δ 9.60 (s, OH), 9.48 (s, OH), 8.62 (d, *J* = 5.0 Hz, 2H), 7.63 (d, *J* = 4.7 Hz, 2H), 7.01 (d, *J* = 6.8 Hz, 2H), 6.78 (d, *J* = 6.9 Hz, 2H), 6.65 (d, *J* = 6.9 Hz, 2H), 6.51 (d, *J* = 6.9 Hz, 2H), 3.21 (t, *J* = 5.0 Hz, 2H), 3.13 (s, 3H), 2.58 (m, 2H), 1.47 (m, 2H).

*4,4′-(5-Methoxy-2-(1-methyl-1H-pyrazol-4-yl)pent-1-ene-1,1-diyl)diphenol* (**12c**). (0.2 g, 91% yield). ^1^H-NMR (400 MHz, DMSO-*d*_6_) δ 7.24 (s, 1H), 6.92 (d, *J* = 6.6 Hz, 2H), 6.79 (d, *J* = 6.6 Hz, 2H), 6.69 (d, *J* = 6.6 Hz, 2H), 6.67 (s, 1H), 6.61 (d, *J* = 6.6 Hz, 2H), 3.68 (s, 3H), 3.21 (t, *J* = 5.1 Hz, 2H), 3.14 (s, 1H), 2.30 (m, 2H), 1.59 (m, 2H).

#### 3.2.8. General Synthetic Procedure for **13a**–**c**

To a solution of **12a**–**c** (1 eq) in acetone/H_2_O (10:1) was added 2-chloro-*N*,*N*-dimethylethylamine hydrochloride (2 eq) and K_2_CO_3_ (2 eq). The mixture was refluxed at 63 °C for 4 h and concentrated under reduced pressure. The crude E/Z mixture (1:1) was purified by preparative HPLC using eluent A (0.1% TFA H_2_O) and eluent B (0.1% TFA ACN) (A/B = 70/30) to give the desired *Z* isomer product.

*(Z)-4-(1-(4-(2-(Dimethylamino)ethoxy)phenyl)-5-methoxy-2-(pyridin-3-yl)pent-1-en-1-yl)phenol* (**13a**). (5 mg, 5% yield). ^1^H-NMR (400 MHz, CD_3_OD) δ 8.23 (d, *J* = 3.1 Hz, 1H), 8.18 (s, 1H), 7.68 (d, *J* = 6.4 Hz, 1H), 7.28 (dd, *J* = 6.2, 4.0 Hz, 1H), 7.16 (d, *J* = 6.9 Hz, 2H), 6.96 (d, *J* = 6.9 Hz, 2H), 6.68 (d, *J* = 6.8 Hz, 2H), 6.46 (d, *J* = 6.8 Hz, 2H), 4.14 (t, *J* = 4.3 Hz, 2H), 3.28 (t, *J* = 5.0 Hz, 2H), 3.21 (s, 3H), 2.81 (t, *J* = 4.3 Hz, 2H), 2.57 (m, 2H), 2.39 (s, 6H), 1.58 (m, 2H). MS *m*/*z* (ESI) 433.2 (M + 1)^+^ (calcd for C_27_H_32_N_2_O_3_H^+^ 432.56).

*(Z)-4-(1-(4-(2-(Dimethylamino)ethoxy)phenyl)-5-methoxy-2-(pyridin-4-yl)pent-1-en-1-yl)phenol* (**13b**). (12 mg, 5% yield). ^1^H-NMR (400 MHz, CD_3_OD) δ 8.32 (d, *J* = 4.9 Hz, 2H), 7.21 (d, *J* = 4.9 Hz, 2H), 7.17 (d, *J* = 6.9 Hz, 2H), 6.99 (d, *J* = 7.0 Hz, 2H), 6.72 (d, *J* = 6.9 Hz, 2H), 6.50 (d, *J* = 6.9 Hz, 2H), 4.18 (t, *J* = 4.3 Hz, 2H), 3.30 (t, *J* = 5.3 Hz, 2H), 3.25 (s, 3H), 2.87 (t, *J* = 4.3 Hz, 2H), 2.60 (m, 2H), 2.43 (s, 6H), 1.60 (m, 2H). MS *m*/*z* (ESI) 433.2 (M + 1)^+^ (calcd for C_27_H_32_N_2_O_3_H^+^ 433.56).

*(Z)-4-(1-(4-(2-(Dimethylamino)ethoxy)phenyl)-5-methoxy-2-(1-methyl-1H-pyrazol-4-yl)pent-1-en-1-yl)-phenol* (**13c**). (3 mg, 2% yield). ^1^H-NMR (400 MHz, CD_3_OD) δ 7.16 (s, 1H), 7.09 (d, *J* = 6.7 Hz, 2H), 6.91 (s, 1H), 6.90 (d, *J* = 6.7 Hz, 2H), 6.85 (d, *J* = 6.6 Hz, 2H), 6.63 (d, *J* = 6.6 Hz, 2H), 4.11 (d, *J* = 4.3 Hz, 2H), 3.73 (s, 3H), 3.35 (m, 2H), 3.25 (s, 3H), 2.80 (t, *J* = 4.2 Hz, 2H), 2.42 (m, 2H), 2.37 (s, 6H), 1.70 (m, 2H). MS *m*/*z* (ESI) 436.3 (M + 1)^+^ (calcd for C_26_H_33_N_3_O_3_H^+^ 436.57).

*(Z)-Methyl 4-bromo-5-(4-(2-(dimethylamino)ethoxy)phenyl)-5-(4-hydroxyphenyl)pent-4-enoate* (**14**). To a solution of **8** (4.1 g, 10.1 mmol) in CH_2_Cl_2_ (80 mL) was added 1M BBr_3_ in CH_2_Cl_2_ (30.3 mL, 30.3 mmol) was added dropwise at 0 °C. The mixture was stirred at rt for 3 h and quenched by adding sat. NaHCO_3_ solution at 0 °C. The mixture was extracted with CH_2_Cl_2_ and extracted with CH_2_Cl_2_. The combined organic layer was concentrated under reduced pressure and crude residue was purified by column chromatography. (2.4 g, 63% yield). To a solution of 2-(dimethylamino)ethanol (0.64 mL, 6.42 mmol) and triphenylphosphine (1.7 g, 6.42 mmol) in CH_2_Cl_2_ (50 mL) stirred at 0 °C. DIAD (1.3 mL, 6.42 mmol) was added dropwise. After 10 min, a solution of methyl 4-bromo-5,5-bis(4-hydroxyphenyl)pent-4-enoate (2.4 g, 6.42 mmol) in CH_2_Cl_2_ (50 mL) was added slowly to the reaction mixture at 0 °C. The reaction mixture was stirred for 12 h at rt and was removed under reduced pressure. The crude *E*/*Z* mixture (1:1) was purified by column chromatography. (0.99 g, 34% yield). ^1^H-NMR (400 MHz, CD_3_OD) δ 7.18 (d, *J* = 8.0 Hz, 2H), 6.98 (m, 4H), 6.72 (d, *J* = 8.0 Hz, 2H), 4.34 (t, *J* = 4.0 Hz, 2H), 3.63 (s, 3H), 3.58 (t, *J* = 4.0 Hz, 2H), 2.98 (s, 6H), 2.87 (m, 2H), 2.62 (m, 2H).

*(E)-Methyl 4-bromo-5-(4-(2-(dimethylamino)ethoxy)phenyl)-5-(4-hydroxyphenyl)pent-4-enoate*. ^1^H-NMR (400 MHz, CD_3_OD) δ 7.13 (d, *J* = 8.0 Hz, 2H), 7.01 (m, 4H), 6.70 (d, *J* = 8.0 Hz, 2H), 4.36 (t, *J* = 4.0 Hz, 2H), 3.63 (s, 3H), 3.59 (t, *J* = 4.0 Hz, 2H), 2.98 (s, 6H), 2.82 (m, 2H), 2.62 (m, 2H).

#### 3.2.9. General Synthetic Procedure for **15a**–**j**

To a solution of **14** (1 eq) in DMF was added corresponding boronic esters or boronic acid (1.5 eq), Pd(dppf)Cl_2_·CH_2_Cl_2_ (0.1 eq) and 2 M Na_2_CO_3_ (3 eq). The mixture was refluxed at 85 °C for 4 h. The reaction mixture was cooled to rt and was quenched by adding water. The mixture was extracted with EtOAc and dried over MgSO_4_. The crude compound was purified by column chromatography. The obtained product was dissolved in THF and added 1 M LiAlH_4_ in THF (1.5 eq) dropwise at 0 °C. The solution was stirred at 0 °C for 1 h and was quenched by adding H_2_O/5 N NaOH/H_2_O (1:1:3). The reaction mixture was filtered off through a Celite pad, washed with EtOAc and concentrated in vacuo. The crude compound was purified by preparative HPLC using eluent A (0.1% TFA H_2_O) and eluent B (0.1% TFA ACN) (A/B = 70/30) to give the desired product.

*(E)-4-(1-(4-(2-(Dimethylamino)ethoxy)phenyl)-5-hydroxypent-1-en-1-yl)phenol (***15a**). (7 mg, 22% yield). ^1^H-NMR (400 MHz, CD_3_OD) δ 7.14 (d, *J* = 8.8 Hz, 2H), 6.94 (d, *J* = 8.4 Hz, 2H), 6.86 (d, *J* = 8.8 Hz, 2H), 6.78 (d, *J* = 8.4 Hz, 2H), 5.94 (t, *J* = 7.2 Hz, 1H), 4.20 (t, *J* = 5.6 Hz, 2H), 3.53 (t, *J* = 6.4 Hz, 2H), 3.16 (t, *J* = 5.2 Hz, 2H), 2.65 (s, 6H), 2.16 (m, 2H), 1.64 (m, 2H). MS *m*/*z* (ESI) 342.2 (M + 1)^+^ (calcd for C_21_H_27_NO_3_H^+^ 342.45).

*(Z)-4,4′-(1-(4-(2-(Dimethylamino)ethoxy)phenyl)-5-hydroxypent-1-ene-1,2-diyl)diphenol* (**15b**). (12 mg, 31% yield). ^1^H-NMR (400 MHz, CD_3_OD) δ 7.15 (d, *J* = 8.4 Hz, 2H), 6.99 (m, 4H), 6.92 (m, 4H), 6.82 (d, *J* = 8.8 Hz, 2H), 6.75 (d, *J* = 8.4 Hz, 2H), 6.66 (m, 4H), 6.58 (m, 4H), 6.43 (d, *J* = 8.8 Hz, 2H), 4.32 (t, *J* = 4.8 Hz, 2H), 4.18 (t, *J* = 5.2 Hz, 2H), 3.47 (m, 2H), 3.40 (m, 6H), 2.89 (s, 6H), 2.84 (s, 6H), 2.46 (m, 4H), 1.55 (m, 4H). MS *m*/*z* (ESI) 434.2 (M + 1)^+^ (calcd for C_27_H_31_NO_4_H^+^ 434.55).

*(Z)-4-(1-(4-(2-(Dimethylamino)ethoxy)phenyl)-5-hydroxy-2-(6-methoxypyridin-3-yl)pent-1-en-1-yl)phenol* (**15c**). (21 mg, 74% yield). ^1^H-NMR (400 MHz, CD_3_OD) δ 7.77 (dd, *J* = 8.9, 1.7 Hz, 2H), 7.51 (dd, *J* = 6.9, 1.9 Hz, 2H), 7.17 (d, *J* = 6.9 Hz, 2H), 7.01 (m, 4H), 6.84 (d, *J* = 7.0 Hz, 2H), 6.77 (d, *J* = 6.8 Hz, 2H), 6.69 (m, 4H), 6.48 (d, *J* = 6.9 Hz, 2H), 4.30 (t, *J* = 4.0 Hz, 2H), 4.17 (t, *J* = 4.0 Hz, 2H), 3.83 (s, 3H), 3.82 (s, 3H), 3.44 (m, 4H), 3.39 (m, 2H), 3.32 (m, 2H), 2.83 (s, 6H), 2.78 (s, 6H), 2.51 (m, 4H), 1.56 (m, 4H). MS *m*/*z* (ESI) 449.2 (M + 1)^+^ (calcd for C_27_H_32_N_2_O_4_H^+^ 449.56).

*(Z)-4-(1-(4-(2-(Dimethylamino)ethoxy)phenyl)-5-hydroxy-2-(pyridin-3-yl)pent-1-en-1-yl)phenol* (**15d**). (2 mg, 19% yield). ^1^H-NMR (400 MHz, CD_3_OD) δ 8.21 (d, *J* = 3.2 Hz, 1H), 8.16 (s, 1H), 7.68 (d, *J* = 5.9 Hz, 1H), 7.28 (d, *J* = 4.2 Hz, 1H), 7.04 (d, *J* = 6.4 Hz, 2H), 6.78 (t, *J* = 7.0 Hz, 4H), 6.64 (d, *J* = 6.6 Hz, 2H), 4.00 (t, *J* = 4.2 Hz, 2H), 3.43 (t, *J* = 5.2 Hz, 2H), 2.81 (t, *J* = 4.0 Hz, 2H), 2.58 (m, 2H), 2.38 (s, 6H), 1.55 (m, 2H). MS *m*/*z* (ESI) 419.2 (M + 1)^+^ (calcd for C_26_H_30_N_2_O_3_H^+^ 419.54).

*(Z)-4-(1-(4-(2-(Dimethylamino)ethoxy)phenyl)-2-(2-fluoropyridin-4-yl)-5-hydroxypent-1-en-1-yl)phenol* (**15e**). (12 mg, 40% yield). ^1^H-NMR (400 MHz, CD_3_OD) δ 7.93 (m, 2H), 7.15 (d, *J* = 8.7 Hz, 2H), 7.03 (m, 4H), 6.96 (d, *J* = 8.7 Hz, 2H), 6.81 (m, 6H), 6.70 (m, 4H), 6.50 (d, *J* = 8.6 Hz, 2H), 4.16 (t, *J* = 5.3 Hz, 2H), 4.04 (t, *J* = 5.3 Hz, 2H), 3.44 (m, 4H), 2.92 (t, *J* = 5.3 Hz, 2H), 2.86 (t, *J* = 5.3 Hz, 2H), 2.58 (m, 4H), 2.46 (s, 6H), 2.42 (s, 6H), 1.54 (m, 4H). MS *m*/*z* (ESI) 437.2 (M + 1)^+^ (calcd for C_26_H_29_FN_2_O_3_H^+^ 437.53).

*(Z)-4-(1-(4-(2-(Dimethylamino)ethoxy)phenyl)-5-hydroxy-2-(6-morpholinopyridin-3-yl)pent-1-en-1-yl)phenol* (**15f**). (7 mg, 57% yield). ^1^H-NMR (400 MHz, CD_3_OD) δ 8.54 (s, OH), 7.76 (d, *J* = 1.7 Hz, 1H), 7.42 (dd, *J* = 7.0, 1.8 Hz, 1H), 7.00 (d, *J* = 6.8 Hz, 2H), 6.84 (d, *J* = 7.0 Hz, 2H), 6.75 (d, *J* = 6.8 Hz, 2H), 6.67 (m, 3H), 4.09 (t, *J* = 4.2 Hz, 2H), 3.75 (t, *J* = 3.7 Hz, 4H), 3.42 (t, *J* = 5.3 Hz, 2H), 3.37 (t, *J* = 3.9 Hz, 4H), 3.07 (t, *J* = 3.7 Hz, 2H), 2.58 (s, 6H), 2.49 (m, 2H), 1.56 (m, 2H). MS *m*/*z* (ESI) 504.3 (M + 1)^+^ (calcd for C_30_H_37_N_3_O_4_H^+^ 504.64).

*(Z)-4-(1-(4-(2-(Dimethylamino)ethoxy)phenyl)-5-hydroxy-2-(1-methyl-1H-indazol-6-yl)pent-1-en-1-yl)phenol* (**15g**). (6 mg, 99% yield). ^1^H-NMR (400 MHz, CD_3_OD) δ 7.87 (s, 1H), 7.50 (d, *J* = 7.6 Hz, 1H), 7.30 (s, 1H), 7.06 (d, *J* = 8.4 Hz, 2H), 6.94 (d, *J* = 8.3 Hz, 1H), 6.83 (d, *J* = 8.7 Hz, 2H), 6.78 (d, *J* = 8.4 Hz, 2H), 6.60 (d, *J* = 8.7 Hz, 2H), 4.06 (t, *J* = 4.6 Hz, 2H), 3.93 (s, 3H), 3.43 (t, *J* = 6.6 Hz, 2H), 3.11 (m, 2H), 2.64–2.61 (m, 7H), 1.57 (m, 2H). ^13^C-NMR (100 MHz, CD_3_OD) δ 157.59, 156.08, 142.01, 140.09, 139.71, 139.01, 136.32, 134.57, 132.03, 131.62, 130.25, 123.53, 122.14, 119.89, 114.54, 113.02, 109.45, 64.11, 61.59, 57.32, 43.90, 33.92, 32.23, 31.75. MS *m*/*z* (ESI) 472.3 (M + 1)^+^ (calcd for C_29_H_33_N_3_O_3_H^+^ 472.60), (Supplementary Materials; Figures S9–S12).

*(Z)-4-(1-(4-(2-(Dimethylamino)ethoxy)phenyl)-5-hydroxy-2-(1-methyl-1H-indol-6-yl)pent-1-en-1-yl)phenol* (**15h**). (3 mg, 6% yield). ^1^H-NMR (400 MHz, CD_3_OD) δ 7.33 (d, *J* = 1.0 Hz, 1H), 7.19 (d, *J* = 8.5 Hz, 2H), 7.14 (d, *J* = 8.5 Hz, 1H), 7.05 (d, *J* = 3.1 Hz, 1H), 7.01 (d, *J* = 8.7 Hz, 2H), 6.91 (dd, *J* = 8.5, 1.5 Hz, 1H), 6.65 (d, *J* = 8.7 Hz, 2H), 6.34 (d, *J* = 8.7 Hz, 2H), 6.28 (d, *J* = 3.0 Hz, 1H), 4.36 (t, *J* = 5.0 Hz, 2H), 3.7 (s, 3H), 3.58 (t, *J* = 5.0 Hz, 2H), 3.38 (t, *J* = 6.8 Hz, 2H), 2.97 (s, 6H), 2.51 (m, 2H), 1.55 (m, 2H). MS *m*/*z* (ESI) 471.3 (M + 1)^+^ (calcd for C_30_H_34_N_2_O_3_H^+^ 471.61).

*(Z)-4-(1-(4-(2-(Dimethylamino)ethoxy)phenyl)-5-hydroxy-2-(imidazo[1,2-a]pyridin-6-yl)pent-1-en-1-yl)phenol* (**15i**). (6 mg, 44% yield). ^1^H-NMR (400 MHz, CD_3_OD) δ 8.55 (s, OH), 8.22 (s, 1H), 7.69 (s, 1H), 7.50 (s, 1H), 7.36 (d, *J* = 8.6 Hz, 1H), 7.12 (d, *J* = 8.9 Hz, 1H), 7.04 (d, *J* = 8.4 Hz, 2H), 6.91 (d, *J* = 8.3 Hz, 2H), 6.78 (d, *J* = 8.4 Hz, 2H), 6.71 (d, *J* = 8.3 Hz, 2H), 4.17 (m, 2H), 3.45 (m, 4H), 2.84 (s, 6H), 2.57 (m, 2H), 1.61 (m, 2H). MS *m/z* (ESI) 458.2 (M + 1)^+^ (calcd for C_28_H_31_N_3_O_3_H^+^ 458.57).

*(Z)-4-(1-(4-(2-(Dimethylamino)ethoxy)phenyl)-5-hydroxy-2-(isoquinolin-7-yl)pent-1-en-1-yl)phenol* (**15j**). (3 mg, 15% yield). ^1^H-NMR (400 MHz, CD_3_OD) δ 9.03 (s, 1H), 8.53 (s, OH), 8.32 (d, *J* = 5.8 Hz, 1H), 7.87 (s, 1H), 7.69 (m, 2H), 7.53 (dd, *J* = 8.5, 1.5 Hz, 1H), 7.08 (d, *J* = 8.6 Hz, 2H), 6.82 (d, *J* = 8.8 Hz, 2H), 6.79 (d, *J* = 8.6 Hz, 2H), 6.58 (d, *J* = 8.8 Hz, 2H), 3.99 (t, *J* = 5.2 Hz, 2H), 3.43 (t, *J* = 6.6 Hz, 2H), 2.90 (t, *J* = 5.2 Hz, 2H), 2.68 (m, 2H), 2.45 (s, 6H), 1.57 (m, 2H). MS *m*/*z* (ESI) 469.2 (M + 1)^+^ (calcd for C_30_H_32_N_2_O_3_H^+^ 469.60).

*(Z)-4-(1-(4-(2-(Dimethylamino)ethoxy)-3-(trifluoromethyl)phenyl)-5-hydroxy-2-(pyridin-3-yl)pent-1-en-1-yl)phenol* (**16**). To a solution of **13a** (66.5 mg, 0.15 mmol) and zinc trifluoromethanesulfinate (127 mg, 0.38 mmol) in CH_2_Cl_2_ (2.5 mL)/H_2_O (1.0 mL) was added TFA (12 μL, 0.15 mmol) and *tert*-butyl hydroperoxide (70% in H_2_O) (64 μL, 0.46 mmol) at 0 °C. The reaction mixture was stirred at rt for 12 h. Upon consumption of the starting material, the reaction was partitioned between CH_2_Cl_2_ (2 mL) and sat. NaHCO_3_ solution (2 mL). The organic layer was separated, and the aqueous layer was extracted with CH_2_Cl_2_ and dried over MgSO_4_. The crude product was purified by preparative HPLC using eluent A (0.1% TFA H_2_O) and eluent B (0.1% TFA ACN) (A/B = 70/30) to give the desired product. (5 mg, 7% yield). ^1^H-NMR (400 MHz, CD_3_OD) δ 8.24 (s, 1H), 8.18 (s, 1H), 7.70 (d, *J* = 6.2 Hz, 1H), 7.28 (m, 3H), 6.94 (d, *J* = 6.7 Hz, 1H), 6.83 (d, *J* = 6.8 Hz, 2H), 6.71 (d, *J* = 6.8 Hz, 2H), 4.11 (t, *J* = 4.0 Hz, 2H), 3.28 (m, 2H), 3.21 (s, 3H), 3.16 (m, 2H), 2.65 (s, 6H), 2.57 (m, 2H), 1.58 (m, 2H). MS *m*/*z* (ESI) 487.2 (M + 1)^+^ (calcd for C_27_H_29_F_3_N_2_O_3_H^+^ 487.54).

*(Z)-4-(1-(4-(2-(Dimethylamino)ethoxy)phenyl)-2-(6-isopropylpyridin-3-yl)-5-methoxypent-1-en-1-yl)phenol* (**17**). To a solution of **13a** (55.9 mg, 0.13 mmol) and zinc isopropylsulfinate (90 mg, 0.32 mmol) in DMSO (1.5 mL) was added TFA (10 μL, 0.13 mmol) and *tert*-butyl hydroperoxide (70% in H_2_O) (54 μL, 0.39 mmol) at 0 °C. The reaction mixture was stirred at rt for 12 h. Upon consumption of the starting material, the reaction was partitioned between EtOAc (5 mL) and sat. NaHCO_3_ solution (5 mL). The organic layer was separated, and the aqueous layer was extracted with EtOAc and dried over MgSO_4_. The crude product was purified by preparative HPLC using eluent A (0.1% TFA H_2_O) and eluent B (0.1% TFA ACN) (A/B = 70/30) to give the desired product. (3 mg, 4% yield). ^1^H-NMR (400 MHz, CD_3_OD) δ 8.04 (s, OH), 7.60 (d, *J* = 6.1 Hz, 1H), 7.17 (d, *J* = 6.1 Hz, 2H), 7.03 (d, *J* = 6.1 Hz, 2H), 6.79 (m, 3H), 6.64 (d, *J* = 6.7 Hz, 2H), 6.45 (d, *J* = 6.4 Hz, 1H), 3.99 (t, *J* = 4.1 Hz, 2H), 3.27 (m, 2H), 3.21 (s, 3H), 2.94 (m, 1H), 2.77 (m, 2H), 2.55 (m, 2H), 2.36 (s, 6H), 1.57 (m, 2H), 1.22 (t, *J* = 5.3 Hz, 6H). MS *m*/*z* (ESI) 475.3 (M + 1)^+^ (calcd for C_30_H_38_N_2_O_3_H^+^ 475.65).

### 3.3. Screening

#### 3.3.1. ERRγ Binding Assay

ERRγ binding assay was conducted with Lantascreen assay system (Life Technologies, Grand Island, NY, USA), which was based on TR-FRET, followed by manufacturer’s instruction. Briefly, compounds were serially diluted with 2-fold staring from 10 μM and 10 μL of each diluent was added in 384 well plate. Then, GST-conjugated ERRγ LBD (ligand-binding domain) was added to be 5 nM. Next, the mixture of fluorescien-conjugated coactivator PGC1a (final concentration to be 500 nM) and Tb-a-GST antibody (final concentration to be 5 nM) was added. The reaction mixture was incubated at rt in dark state for 1 h. TR-FRET activity was measured at 340 nm excitation and 495 nm/520 nm dual emission using microplate reader (Biotek, SynergyNeo, Winooski, VT, USA) and IC_50_ value was calculated with the Prism 6 software [15,16,17].

#### 3.3.2. ERRα, ERRβ, ERα Binding Assay

ERRs and ERα binding assay used same assay system as ERRγ’s, Lanthascreen assay system. Therefore, procedure was same as ERRγ binding assay except tested enzymes (ERRα, ERRβ, ERα). Used reference compounds were 4-OHT 1 for ERα and ERRβ binding assay, and XCT790 for ERRα binding assay.

#### 3.3.3. ERRγ Functional Assay

AD293 cells were cultured in Dulbecco’ modified Eagle medium (DMEM) High glucose supplemented with 0.5% FBS (Hyclone, Logan, UT, USA). After 24 h, culture media was changed with DMEM High glucose containing 10% FBS. DNA constructs, which includes pCMX-Gal4-ERRγ, pFR-luciferase reporter plasmid, pCMV-β-gal were transiently transfected into AD293 cells using TransIT-LT1 transfection reagent (Mirus, Madison, WI, USA) for 24 h. Then, transfected cells were treated with compounds for 24 h and harvested for detection of luciferase activity and β-gal activity. All cells were incubated at 37 °C under 5% CO_2_ in a humidified incubator [5].

#### 3.3.4. Metabolic Stability Assay in Liver Microsomes

The metabolic stability assay was performed by incubation of human and selected animal liver microsomes (most often dog, rat and mouse), at 37 °C with a test compound at a final concentration of 1 μM, in the presence of 0.5 mg/mL microsomal protein and NADPH regeneration system, in a total volume of 100 μL of 100 mM phosphate buffer, pH 7.4. The incubation was started by the addition of NADPH regeneration system and terminated with adding 40 μL of ice-cold acetonitrile at 0 and 30 min. Precipitated proteins were removed by centrifugation at 10,000× *g* for 5 min at 4 °C. Aliquots of the supernatant were injected onto an LC-MS/MS system. Incubations terminated prior to addition of NADPH regeneration system (time point 0 min) were used as standards, defined as 100%. Percent of the parent compound remaining was calculated by comparing peak areas [18].

#### 3.3.5. CYP Inhibition Assay

All incubations were performed in duplicate, and the mean values were used for analysis. The assays of phenacetin *O*-deethylase, tolbutamide 4-hydroxylase, *S*-mephenytoin 4-hydroxylase, dextromethorphan *O*-demethylase and midazolam 1′-hydroxylase activities were determined as probe activities for CYP1A2, CYP2C9, CYP2C19, CYP2D6 and CYP3A, respectively, using cocktail incubation and tandem mass spectrometry. Briefly, incubation reaction was performed with 0.25 mg/mL human liver microsomes in a final incubation volume of 100 μL. The incubation medium contained 100 mM phosphate buffer (pH 7.4) with probe substrates. The incubation mixture containing various inhibitors (10 μM) was pre-incubated for 5 min. After pre-incubation, an NADPH regenerating system was added. After incubation at 37 °C for 15 min, the reaction was stopped by placing the incubation tubes on ice and adding 40 μL of ice-cold acetonitrile. The incubation mixtures were then centrifuged at 10,000× *g* for 5 min at 4 °C. Aliquots of the supernatant were injected onto an LC-MS/MS system. The CYP-mediated activities in the presence of inhibitors were expressed as percentages of the corresponding control values [19].

#### 3.3.6. hERG Assays

hERG channel binding assay was performed using predictor hERG fluorescence polarization assay (Cat.No. PV5365, Invitrogen, Grand Island, NY, USA) according to the manufacturer’s instructions. Briefly, for measuring IC_50_, compounds were serially diluted (16 points, 3-fold) and then followed by reactions for 4 h at 25 °C in a reaction mixture containing hERG membrane, fluorescence tracer red dye and fluorescence polarization buffer. Fluorescence intensity (Excitation at 530 nm, Emission at 590 nm) was measured using a multi-mode microplate reader Synergy Neo (Biotek, Winooski, VT, USA). E-4031 was used as the reference positive standard (IC_50_ = 10–90 nM) [20].

### 3.4. Molecular Docking Analysis of ERRγ Inverse Agonists

For the prediction of docking model of compound **15g** with ERRγ, we used the crystal structure of human ERRγ and its complex with **4** from Protein Data Bank (PDB code: 2EWP). The protein structure was minimized using the Protein Preparation Wizard by applying an OPLS force field. After complete preparation of the ligands and protein for docking, receptor-grid files were generated. Ligand docking into the active site of ERRγ was carried out using the Schrödinger docking program, Glide. The energy minimized compound **15g** was docked into the prepared receptor grid. The best-docked pose was selected as the lowest Glide score. The molecular graphics for the agonist binding pocket and refined docking model for the selected compounds was generated using PyMol [21].

## 4. Conclusions

In this report, we describe the discovery and structural exploration of new analogs of compound **4** as ERRγ inverse agonists. The A-ring in the **4** scaffold was mainly modified to generate drug candidates that were then tested using *in vitro* binding and functional screening assays. Among the compounds tested, compound **15g** represents a new structural class of promising **4** analogs that are fully subtype selective ERRγ inverse agonists. In addition, these compounds have acceptable ADMET profiles and show potential for the treatment of a variety of ERRγ-related disorders. Additional studies analyzing compound **15g** in animal disease models are underway.

## Supplementary Materials

Supplementary materials can be accessed at: http://www.mdpi.com/1420-3049/21/1/80/s1.

## Abbreviations

ERRγ: Estrogen-Related Receptor gamma
ADMET: Absorption, distribution, metabolism, excretion, toxicity
hERG: Human ether-a-go-go related gene
ERRs: Estrogen-related receptors
ER: Estrogen receptors
CYP: Cytochrome p450
NIS: Sodium iodide symporter

## References

[1] Tremblay A.M., Giguere V. (2007). The NR3B subgroup: An ovERRview. Nucl. Recept. Signal..

[2] Heard D.J., Norby P.L., Holloway J., Vissing H. (2000). Human ERRgamma, a third member of the estrogen receptor-related receptor (ERR) subfamily of orphan nuclear receptors: Tissue-specific isoforms are expressed during development and in the adult. Mol. Endocrinol..

[3] Ariazi E.A., Jordan V.C. (2006). Estrogen-related receptors as emerging targets in cancer and metabolic disorders. Curr. Top. Med. Chem..

[4] Kim D.K., Gang G.T., Ryu D., Koh M., Kim Y.N., Kim S.S., Park J., Kim Y.H., Sim T., Lee I.K. (2013). Inverse agonist of nuclear receptor ERRgamma mediates antidiabetic effect through inhibition of hepatic gluconeogenesis. Diabetes.

[5] Kim D.K., Ryu D., Koh M., Lee M.W., Lim D., Kim M.J., Kim Y.H., Cho W.J., Lee C.H., Park S.B. (2012). Orphan nuclear receptor estrogen-related receptor gamma (ERRgamma) is key regulator of hepatic gluconeogenesis. J. Biol. Chem..

[6] Kim D.K., Kim J.R., Koh M., Kim Y.D., Lee J.M., Chanda D., Park S.B., Min J.J., Lee C.H., Park T.S. (2011). Estrogen-related receptor gamma (ERRgamma) is a novel transcriptional regulator of phosphatidic acid phosphatase, LIPIN1, and inhibits hepatic insulin signaling. J. Biol. Chem..

[7] Kim D.K., Jeong J.H., Lee J.M., Kim K.S., Park S.H., Kim Y.D., Koh M., Shin M., Jung Y.S., Kim H.S. (2014). Inverse agonist of estrogen-related receptor gamma controls Salmonella typhimurium infection by modulating host iron homeostasis. Nat. Med..

[8] Coward P., Lee D., Hull M.V., Lehmann J.M. (2001). 4-Hydroxytamoxifen binds to and deactivates the estrogen-related receptor gamma. Proc. Natl. Acad. Sci. USA.

[9] Nam K., Marshall P., Wolf R.M., Cornell W. (2003). Simulation of the different biological activities of diethylstilbestrol (DES) on estrogen receptor alpha and estrogen-related receptor gamma. Biopolymers.

[10] Zuercher W.J., Gaillard S., Orband-Miller L.A., Chao E.Y., Shearer B.G., Jones D.G., Miller A.B., Collins J.L., McDonnell D.P., Willson T.M. (2005). Identification and structure-activity relationship of phenolic acyl hydrazones as selective agonists for the estrogen-related orphan nuclear receptors ERRbeta and ERRgamma. J. Med. Chem..

[11] Chao E.Y., Collins J.L., Gaillard S., Miller A.B., Wang L., Orband-Miller L.A., Nolte R.T., McDonnell D.P., Willson T.M., Zuercher W.J. (2006). Structure-guided synthesis of tamoxifen analogs with improved selectivity for the orphan ERRgamma. Bioorg. Med. Chem. Lett..

[12] Singh T.D., Jeong S.Y., Lee S.W., Ha J.H., Lee I.K., Kim S.H., Kim J., Cho S.J., Ahn B.C., Lee J. (2015). Inverse Agonist of Estrogen-Related Receptor gamma Enhances Sodium Iodide Symporter Function through Mitogen-Activated Protein Kinase Signaling in Anaplastic Thyroid Cancer Cells. J. Nucl. Med..

[13] Guthrie R.W., Kierstead R.W., Mullin J.G., Tilley J.W. (1990). Preparation of heterocyclic (especially pyridine) compounds useful in treating diseases characterized by excess platelet activating factor (PAF). U.S. Patent.

[14] Fujiwara Y., Dixon J.A., Rodriguez R.A., Baxter R.D., Dixon D.D., Collins M.R., Blackmond D.G., Baran P.S. (2012). A new reagent for direct difluoromethylation. J. Am. Chem. Soc..

[15] Zhou G., Cummings R., Li Y., Mitra S., Wilkinson H.A., Elbrecht A., Hermes J.D., Schaeffer J.M., Smith R.G., Moller D.E. (1998). Nuclear receptors have distinct affinities for coactivators: Characterization by fluorescence resonance energy transfer. Mol. Endocrinol..

[16] Puigserver P., Wu Z., Park C.W., Graves R., Wright M., Spiegelman B.M. (1998). A cold-inducible coactivator of nuclear receptors linked to adaptive thermogenesis. Cell.

[17] Wu X., Glickman J.F., Bowen B.R., Sills M.A. (2003). Comparison of assay technologies for a nuclear receptor assay screen reveals differences in the sets of identified functional antagonists. J. Biomol. Screen..

[18] Lu C., Li P., Gallegos R., Uttamsingh V., Xia C.Q., Miwa G.T., Balani S.K., Gan L.S. (2006). Comparison of intrinsic clearance in liver microsomes and hepatocytes from rats and humans: Evaluation of free fraction and uptake in hepatocytes. Drug Metab. Dispos..

[19] Otten J.N., Hingorani G.P., Hartley D.P., Kragerud S.D., Franklin R.B. (2011). An *in vitro*, high throughput, seven CYP cocktail inhibition assay for the evaluation of new chemical entities using LC-MS/MS. Drug Metab. Lett..

[20] Piper D.R., Duff S.R., Eliason H.C., Frazee W.J., Frey E.A., Fuerstenau-Sharp M., Jachec C., Marks B.D., Pollok B.A., Shekhani M.S. (2008). Development of the predictor HERG fluorescence polarization assay using a membrane protein enrichment approach. Assay Drug Dev. Technol..

[21] PyMOL v1.7.6. http://www.pymol.org.

