# Supplementary material for: Identification of Selective ERRγ Inverse Agonists
Source: Molecules. 2016 Jan 12;21(1):80. doi: 10.3390/molecules21010080 (PMC6273807; doi:10.3390/molecules21010080)
Supplement: Supplementary file 1 [file molecules-21-00080-s001.pdf]

Supplementary Materials: Identification of Selective ERRγ Inverse Agonists

Jina Kim, Chun Young Im, Eun Kyung Yoo, Min Jung Ma, Sang-Bum Kim, Eunmi Hong, Jungwook Chin, Hayoung Hwang, Sungwoo Lee, Nam Doo Kim, Jae-Han Jeon, In-Kyu Lee, Yong Hyun Jeon, Hueng-Sik Choi, Seong Heon Kim and Sung Jin Cho

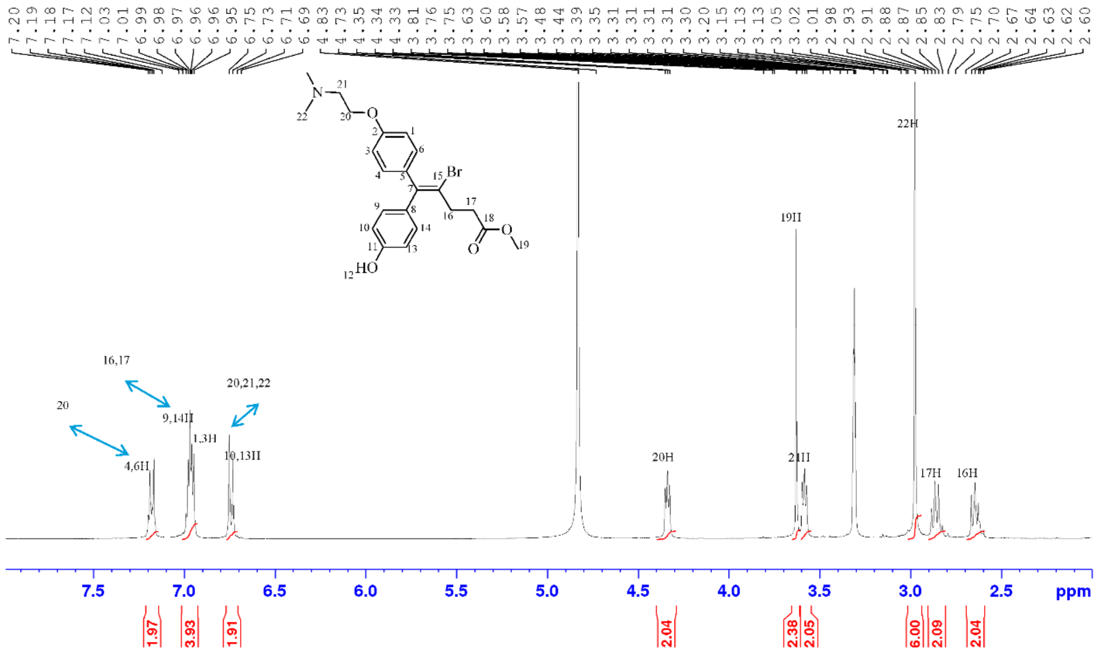

Figure S1. ^{1}H-NMR spectrum of compound **14**.

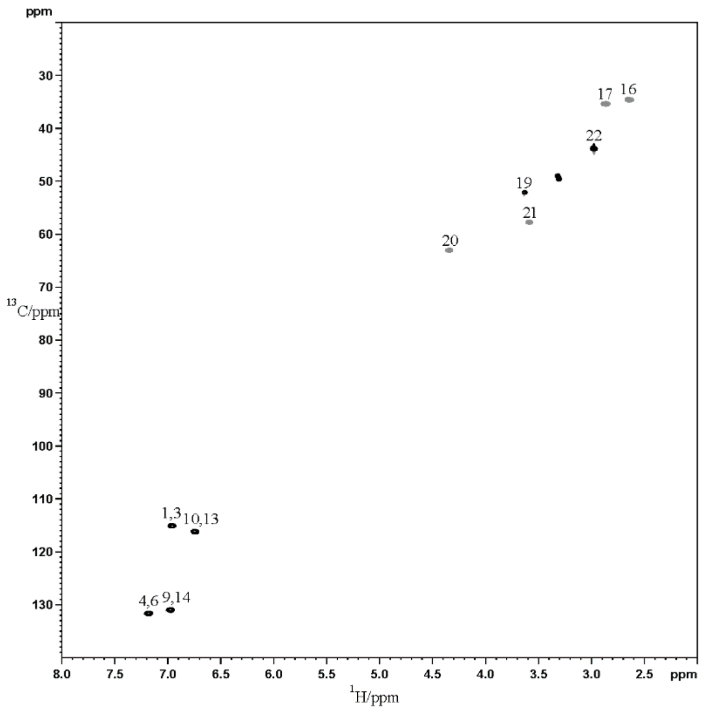

Figure S2. HSQC spectrum of compound **14**.

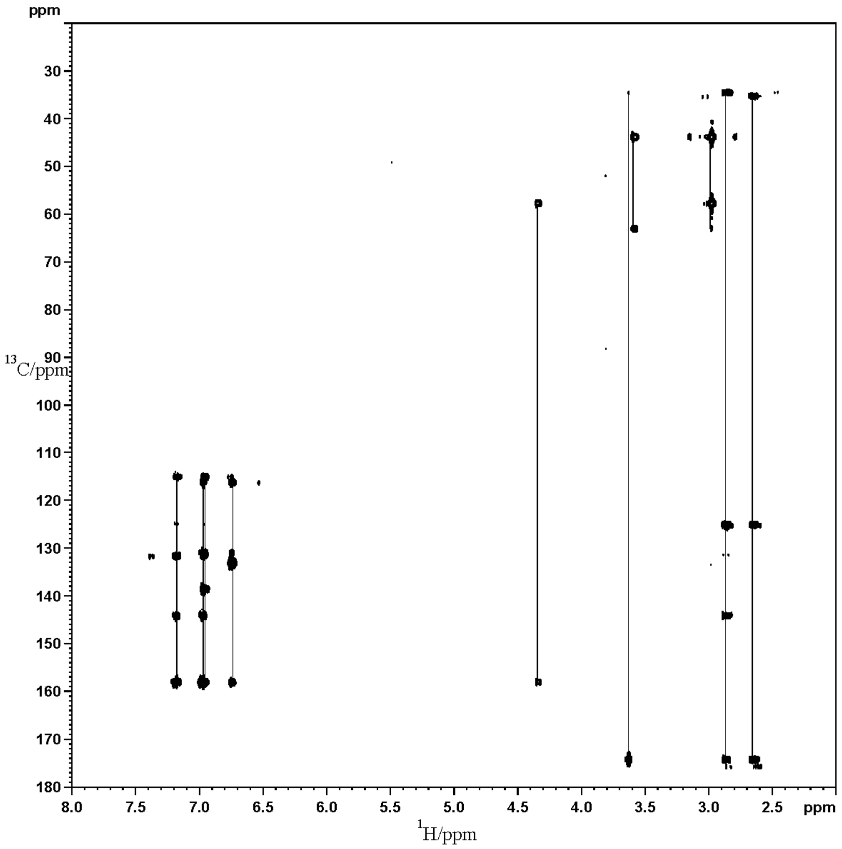

Figure S3. HMBC spectrum of compound **14**.

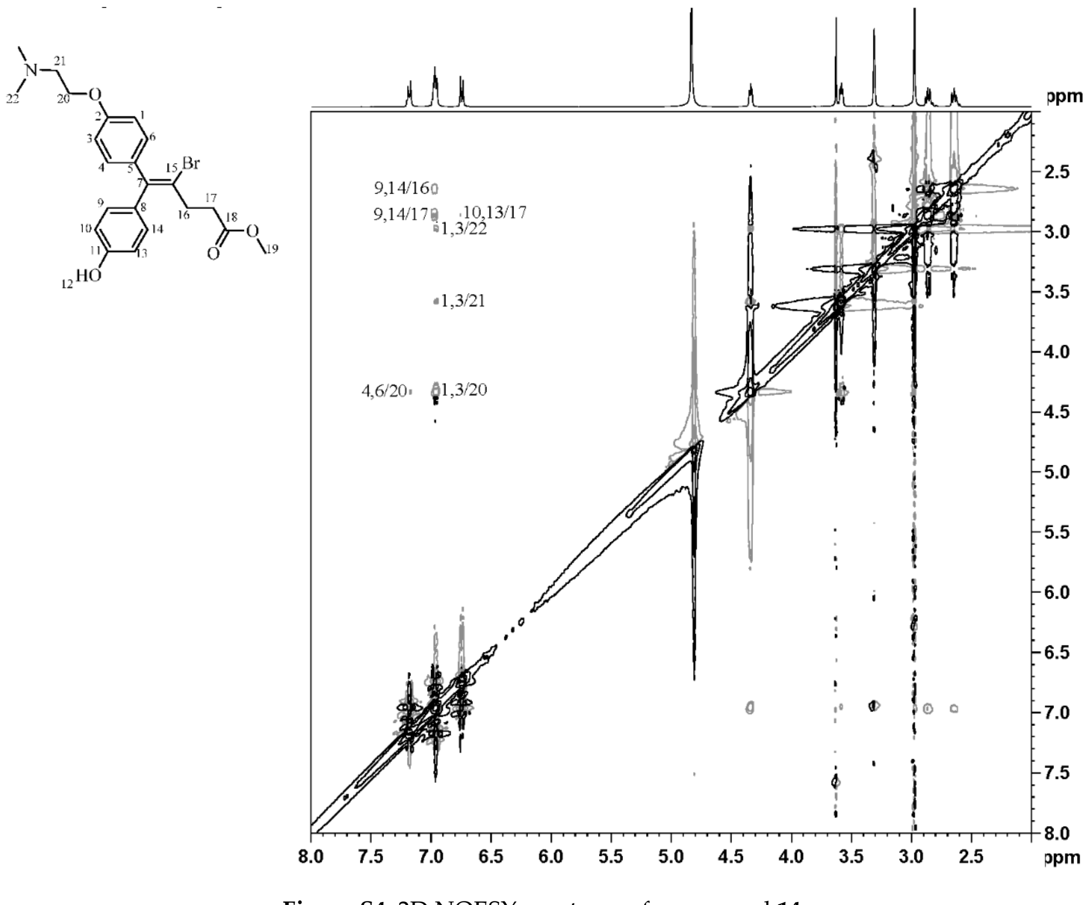

Figure S4. 2D NOESY spectrum of compound **14**.

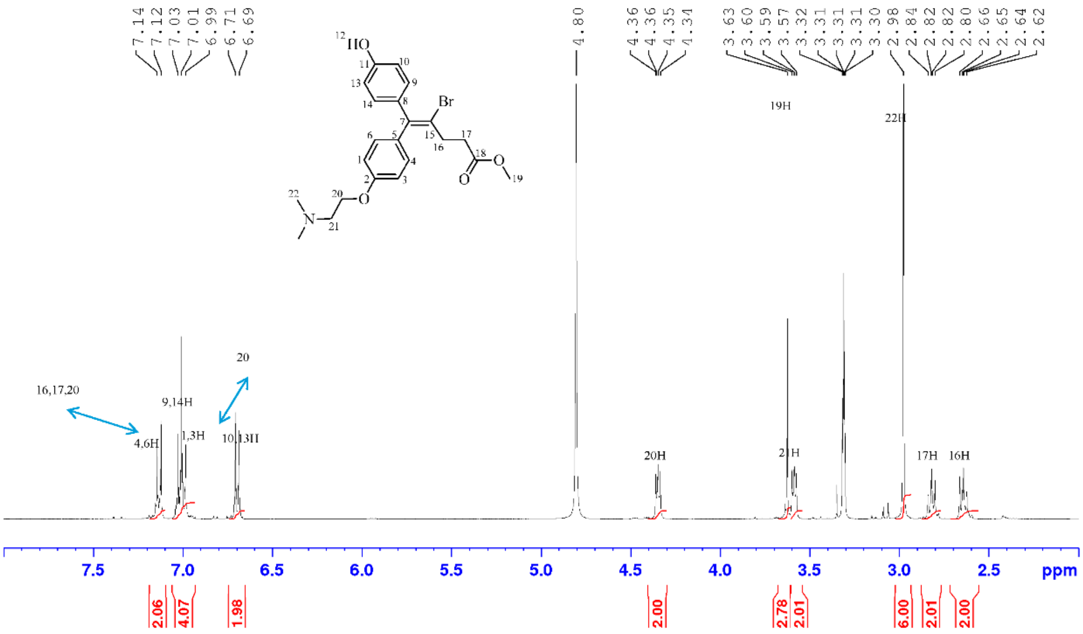

Figure S5. ^{1}H-NMR spectrum of E isomer of compound **14**.

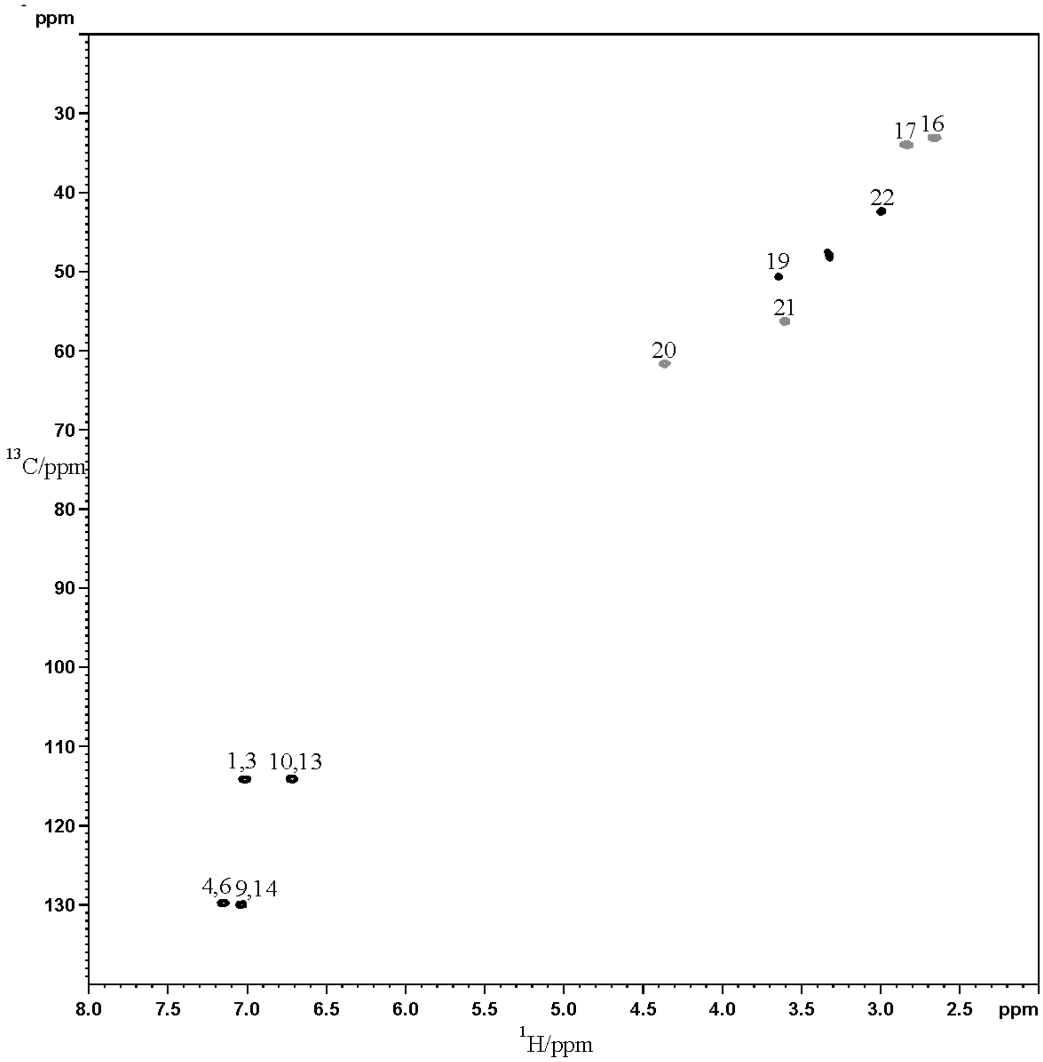

Figure S6. HSQC spectrum of E isomer of compound **14**.

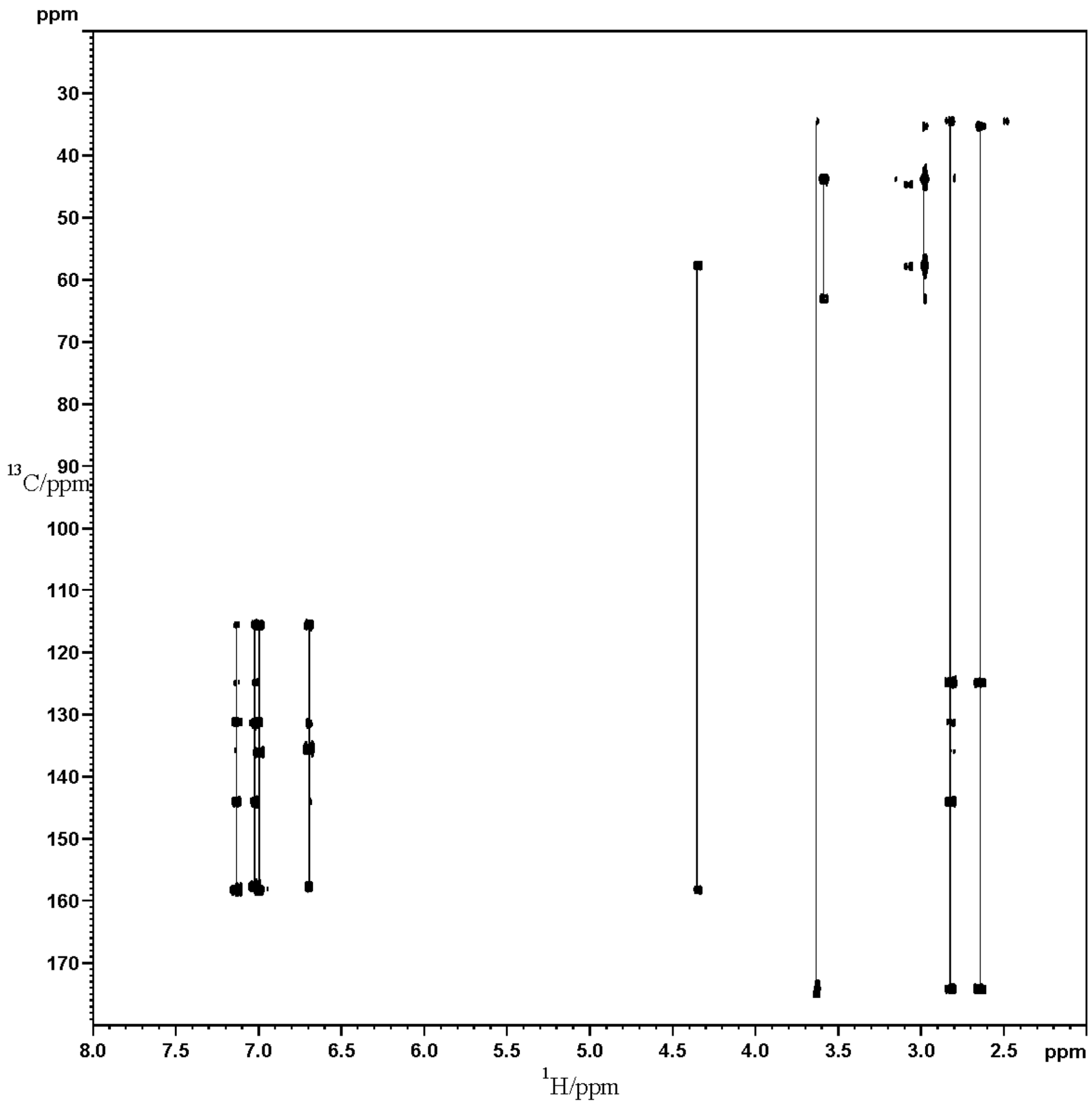

Figure S7. HMBC spectrum of E isomer of compound **14**.

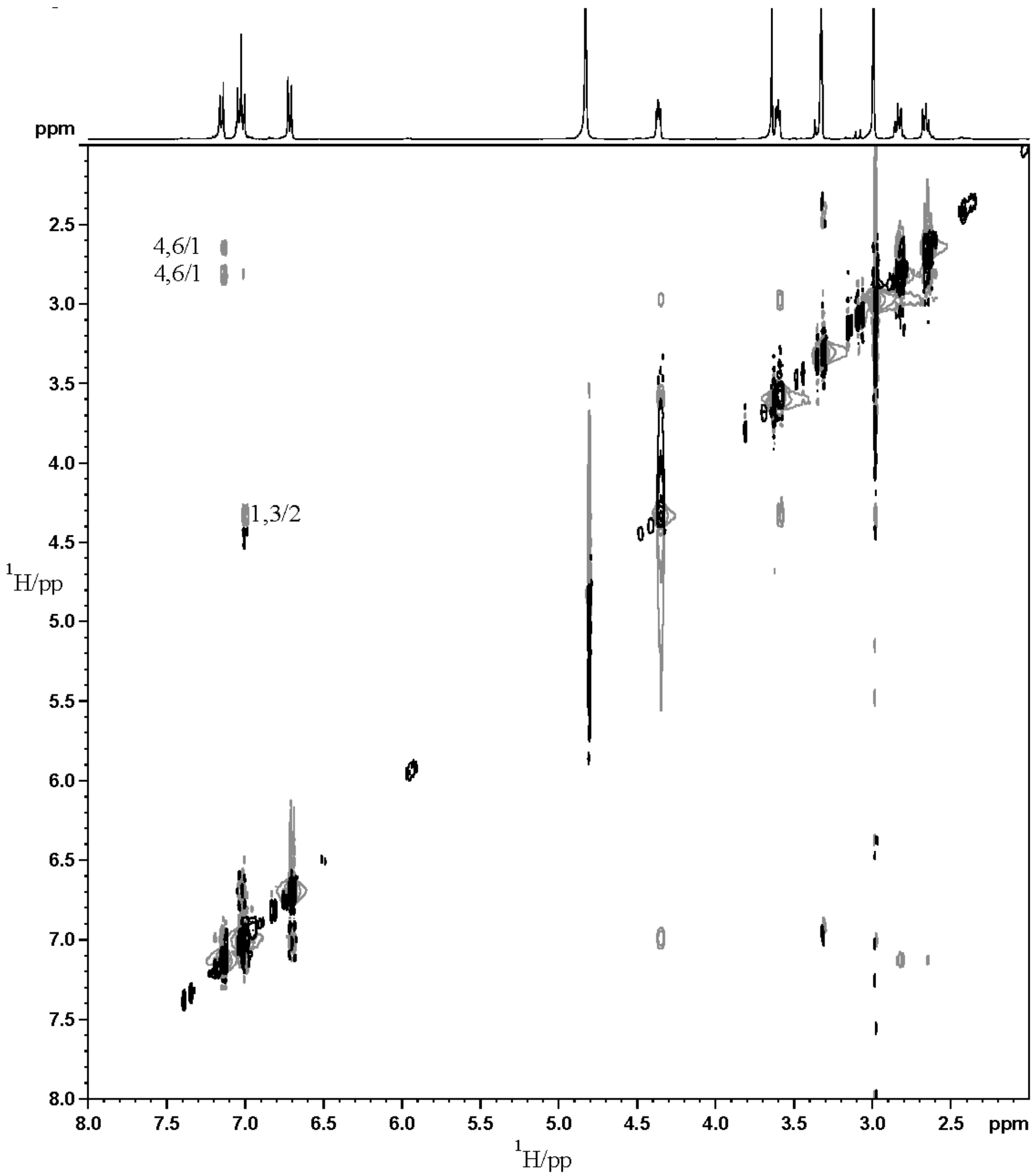

Figure S8. 2D NOESY spectrum of E isomer of compound **14**.

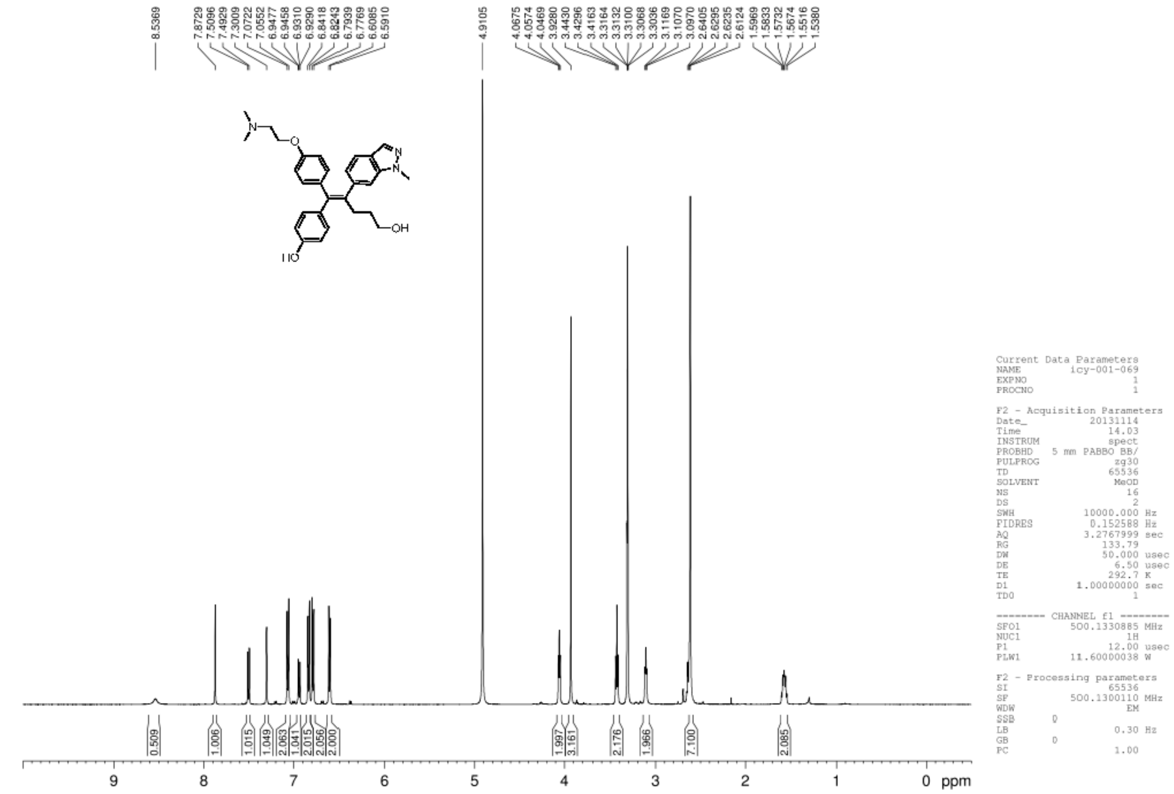

Figure S9. ^{1}H-NMR spectrum of compound **15g**.

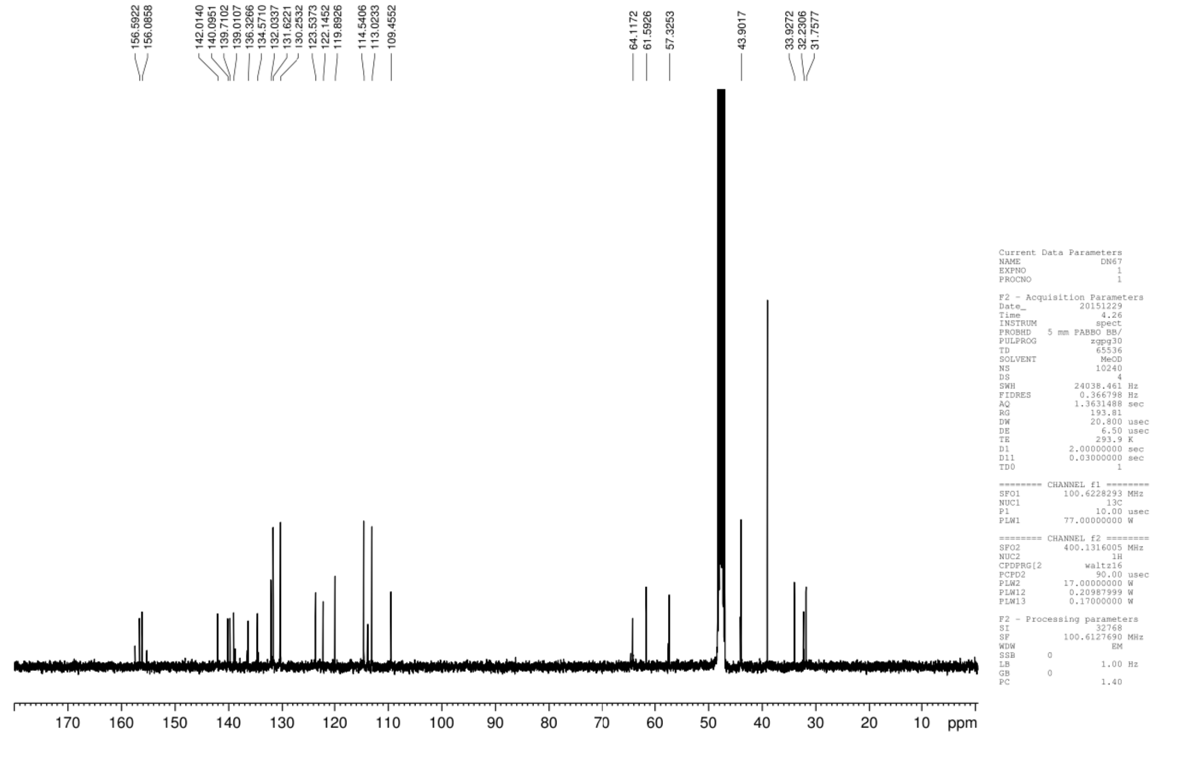

Figure S10. ^{13}C-NMR Spectrum of compound **15g**.

Operator:n.a. Timebase:U-3000 Sequence:ICY-002-006 Page 1-1
2014-7-17 오후 14:18

11 ICY-002-006final

Sample Name:	**ICY-002-006final**	*Injection Volume:*	**5.0**
Vial Number:	**GA1**	*Channel:*	**UV_VIS_1**
Sample Type:	**Unknown**	*Wavelength:*	**n.a.**
Control Program:	**cyim**	*Bandwidth:*	**n.a.**
Quantif. Method:	**cyim**	*Dilution Factor:*	**1.0000**
Recording Time:	**2014-4-23 14:55**	*Sample Weight:*	**1.0000**
Run Time (min):	**10.00**	*Sample Amount:*	**1.0000**

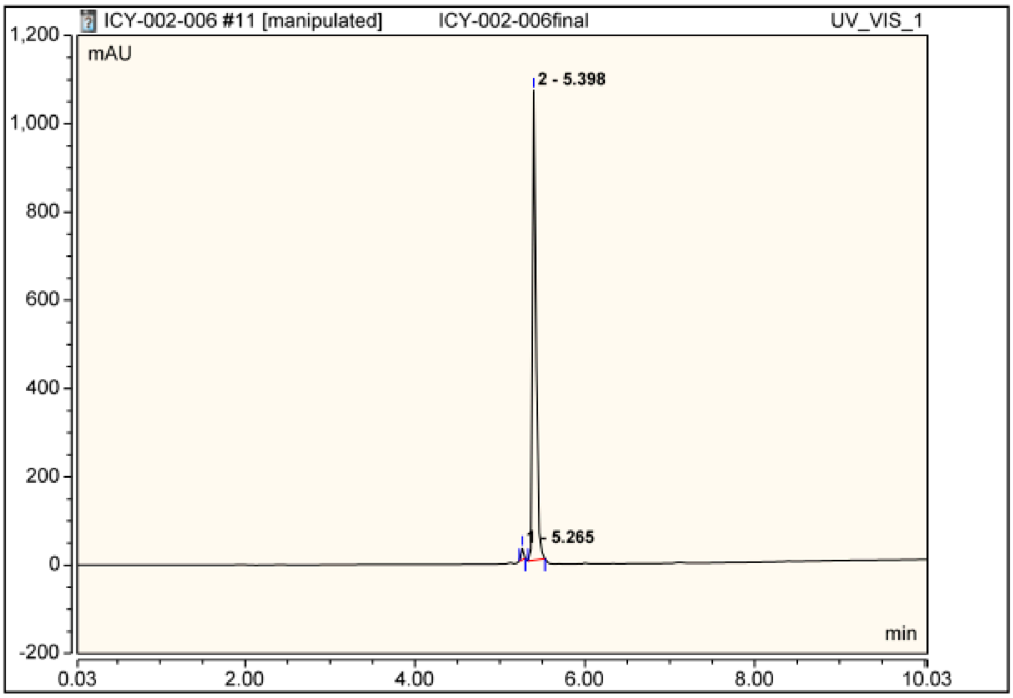

No.	Ret.Time min	Peak Name	Height mAU	Area mAU*min	Rel.Area %	Amount n.a.	Type
1	5.26		25.268	0.843	1.56	n.a.	BMB*
2	5.40		1064.211	53.270	98.44	n.a.	BMB*
**Total:**			1089.480	54.113	100.00	0.000	

Figure S11. HPLC Spectrum of compound **15g**.

==== Shimadzu LabSolutions Browser Report ====

Sample Information(icy-002-006final.lcd)

**Data File Name:**	**icy-002-006final.lcd**
**Method File Name:**	**2014.2.5_new install test.lcm**
**Acquired by:**	**System Administrator**
**Date Acquired:**	**2014-04-23 2:25:47**
**Sample Name:**	**1**
**Sample ID:**	**1**
**Sample Type:**	**Unknown**
**Level#:**	**0**
**Detector:**	**PDA, MS**
**Comment:**	

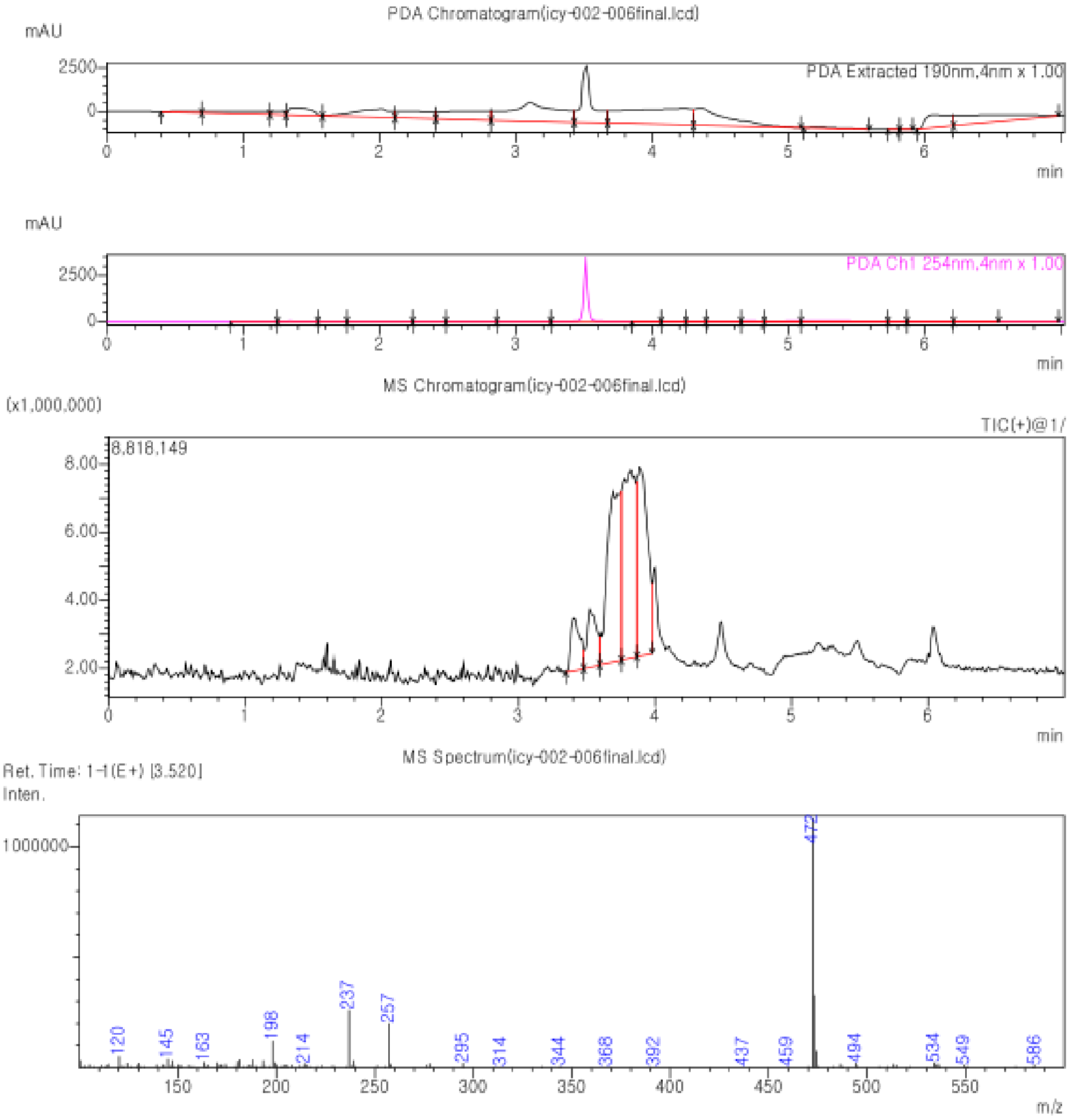

Figure S12. LC/MS Spectrum of compound **15g**.
